# Lead, Locked Away: Porous Zr–Phytate Coordination Polymers for Rapid and Selective Removal of Pb^2+^ from Water

**DOI:** 10.1021/jacs.5c11825

**Published:** 2025-12-04

**Authors:** Nazanin Taheri, Till Schertenleib, Timo M. O. Felder, Laura Piveteau, Beatriz Mouriño, Berend Smit, Mohammad Khaja Nazeeruddin, Wendy L. Queen

**Affiliations:** † Laboratory for Functional Inorganic Materials (LFIM), Institute of Chemical Sciences and Engineering (ISIC), 27218 École Polytechnique Fédérale de Lausanne (EPFL), Sion 1951, Switzerland; ‡ Nuclear Magnetic Resonance Platform (NMRP), Institute of Chemical Sciences and Engineering (ISIC), École Polytechnique Fédérale de Lausanne (EPFL), Lausanne 1015, Switzerland; § Laboratory of Molecular Simulations (LSMO), Institute of Chemical Sciences and Engineering (ISIC), École Polytechnique Fédérale de Lausanne (EPFL), Sion 1951, Switzerland; ∥ Institute of Chemical Sciences and Engineering (ISIC), École Polytechnique Fédérale de Lausanne (EPFL), Lausanne 1015, Switzerland

## Abstract

Coordination polymers (CPs) often suffer from poor hydrolytic and chemical stability, limiting their use in water remediation. Herein, we report a highly robust, amorphous CP synthesized from Zr^4+^ and phytic acid, a natural source of phosphorus in plants and seeds. The CP, which features stable Zr–O–P bonds that resist degradation even in 10 M HCl and HNO_3_, forms a micro- and mesoporous network under mild reaction conditions in water. The material consists of mononuclear ZrO_6_ units bridged by phosphate groups. Zr–Phytate exhibits excellent Pb^2+^ removal performance, maintaining high efficiency even in the presence of excess competing ions. Pair Distribution Function (PDF) analysis and solid-state NMR (ssNMR) provide insight into the coordination environment of the Zr-phosphate centers and the mechanism of lead complexation. The exceptional chemical durability of Zr–Phytate allows efficient regeneration using 1 M HCl, with no detectable leaching of Zr or phytic acid and no loss of structural integrity over multiple cycles. Compared to commercial ion-exchange resins, Zr–Phytate offers superior selectivity and reusability. This work demonstrates the importance of designing stable coordination polymers and highlights the promise of zirconium-phosphate networks for applications in water remediation.

## Graphical abstract

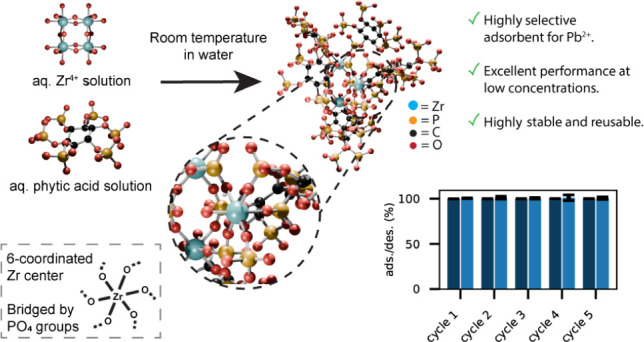

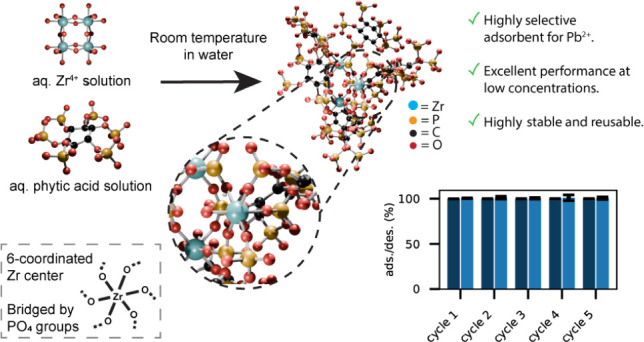

## Introduction

Lead contamination in water, a global health crisis across low- and high-income regions, is driven by inadequate treatment and rising industrial discharge; in many developing countries, up to 80% of industrial wastewater is discharged into surrounding water sources untreated.^1–3^ Lead (Pb^2+^) is especially pernicious—persistent in ecosystems and presenting the risk of irreversible neurological damage at parts-per-billion levels—leading the WHO to designate it as a top chemical of critical public health concern.^4–7^ Several approaches to remediation exist: chemical precipitation,^8^ membrane processes,^9^ biological treatment,^10^ ion exchange,^11^ adsorption,^12^ and photocatalytic oxidation.^13^ Among these, adsorption stands out as particularly advantageous due to its cost-effectiveness, operational simplicity, minimal energy requirements, and potential for pollutant recovery rather than mere phase transfer.^14–16^ Ideal adsorbents would enable the removal of large quantities of targeted species and be cheap, selective, scalable, and stable in environmentally relevant conditions.^17,18^ From a material development perspective, this means that materials should contain high densities of accessible adsorption sites and have a high affinity for the target species.^18^ Ideally, these materials should also be synthesized using low energy input, employ cheap and nontoxic reagents, and be regenerable.

Porous crystalline coordination polymers (CPs), like metal–organic frameworks (MOFs) have gained much attention as adsorbents for water purification.^19–22^ Most MOFs are highly porous, and they can be designed with specific pore shapes, sizes, and surface functionality.^21,23^ This modular nature allows the design of structures having high densities of well-defined, accessible adsorption sites, providing direct insight into how to optimize adsorbent performance toward a targeted analyte. As an example, Yu et al. developed a MOF composed of Zn-paddlewheel metal nodes interlinked with 4,4’-azoxydibenzoic acid, containing polarized negatively charged oxygen groups as adsorption sites for Pb^2+^.^24^ The MOF had high Pb^2+^ selectivity, a high maximum adsorption capacity of *q*_e_ = 616 mg/g, and a high affinity toward Pb^2+^ at low concentration, determined by the distribution constant *K*_d_ ≈ 2,300 L/g at an equilibrium concentration of *c*_e_ = 35 ppb (corresponding to a *q*_e_ of approximately 80 mg/g). A host of other MOFs have been explored for the removal of Pb^2+^ from aqueous environments. For example, several benzene dicarboxylate (BDC) and benzene tricarboxylate (BTC)-linked MOFs, including Al-BDC,^25^ Cu-BTC,^26^ and Zn-BTC,^26^ were found to offer adsorption capacities of 429, 333, and 312 mg Pb^2+^ per g of adsorbent, respectively.

While the performance metrics of these MOFs and others^12,27,28^ compare well against commercial resins and carbons, several drawbacks limit their implementation in water treatment applications. First, many MOFs are synthesized using toxic solvents such as DMF, limiting their scalability^29^ and applicability in water treatment. Second, many MOFs, including the examples listed above^24–26^ and in particular first-row transition metal-based CPs, are not stable enough to withstand hydrolysis after long-term exposure to water.^30^ Third, MOFs are often not stable under the harsh conditions required for regeneration. For example, while some effort was made to assess the cyclability of Zn-4,4’-azoxydibenzoate, experimental proof of Pb^2+^ desorption was missing. Further, the MOF’s structural integrity after cycling was also not evaluated.

The material with the highest Pb^2+^ adsorption capacity we have identified is a defective Zr-2,6-naphthalenedicarboxylate-based MOF rich in O-containing adsorption sites.^31^ The reported MOF has a *q*_max_ of 1309 mg/g and shows only minimal loss in Pb^2+^ removal efficiency after 7 adsorption–desorption cycles. However, the experimental conditions are insufficiently documented to understand whether Pb^2+^ was effectively desorbed between each cycle. Furthermore, the reported PXRD data clearly indicate a loss of crystallinity after just one cycle. While the loss of crystallinity on its own is not an issue if the material retains its function, such structural changes should not lead to secondary pollution due to leaching of inorganic or organic building blocks.

These examples demonstrate a fundamental limitation of carboxylate-based MOFs for water purification: their limited stability in aqueous media or the conditions used to regenerate the material.^30^ To address this issue, we have begun working to construct more robust materials that feature stronger metal–ligand bonds. As inspiration, we have turned to Zr-phosphate chemistry, which we believe offers particular promise. Notably, purely inorganic Zr-phosphate salts, referred to as ZrP, are practically water-insoluble and possess unique properties as proton conductors and ion-exchangers, albeit they have limited porosity.^32–35^ To overcome the latter, one can make hybrid materials using organic phosphate and phosphonate ligands as rigid struts. Such building blocks not only boost porosity but are also known to form exceptionally strong bonds with high-valent group IV metal ions.^36,37^ This robust chemistry has gained increasing attention in porous materials research^38–41^ with several crystalline Zr-phosphonate MOFs demonstrating sufficient stability for uranium recovery from highly acidic nuclear waste streams.^42^

Enhanced stability often comes with certain trade-offs. For example, strong metal–ligand bonds that provide necessary aqueous stability can also reduce bond lability, which is needed for the formation of long-range order and thus crystalline structures. Thus, such ligands often instead result in poorly crystalline or amorphous CPs.^36,37^ From previous reports, we ascertain that the formation of crystalline Zr-phosphonates requires quite harsh conditions—HF modulators (since fluoride competes with Zr-phosphate bond formation)^42,43^ and/or refluxing in phosphoric acid^44^ —increasing production costs and environmental impact without clear performance benefits. In contrast, amorphous Zr-phosphonate sorbents offer compelling advantages for water purification. They can be synthesized under mild conditions^37^ requiring significantly less energy and caustic materials than crystalline structures^45,46^ while accommodating diverse organic ligands, including flexible bioavailable linkers.^47^ While structural characterization is certainly more challenging for amorphous CPs, and they may not be as porous as their crystalline counterparts (namely, MOFs), it is unclear if such factors will impede their overall performance. This inspired us to explore the formation of amorphous Zr-CPs for practical water remediation applications.

In this context, phytic acid is an interesting organic building block for synthesizing micro- and mesoporous Zr-CPs, owing to the fact that it is a strong metal chelate^48–51^ and a naturally occurring biomolecule that is a source of phosphorus in plants and seeds.^52^ The advantage of using phytic acid instead of a simple phosphate is that it may promote the formation of less-dense porous structures and provide excess phosphate groups that remain unbound to Zr, which can boost the metal adsorption capacity and kinetics during adsorption. Notably, phosphates have four oxygen groups that can bind to metals, even in their protonated state,^36^ and the number of available phosphate groups unbound to Zr will dictate the ability of the material to remove toxic metals from solution.

Considering these advantages, we report the fabrication of Zr–Phytate CPs (Figure 1), which have led to the discovery of an economical and environmentally friendly material that offers impressive performance in Pb^2+^ removal from aqueous streams. The Zr–Phytate CP retained a considerable number of uncoordinated phosphate groups, enabling it to readily bind Pb^2+^ ions, thereby boosting its capacity in the low concentration regime relevant to water remediation. When compared to several top-performing commercial resins, Zr–Phytate showed exceptionally high selectivity for Pb^2+^, which allowed the material to retain high removal efficiency even with competing ions present in a 100-fold excess. Owing to its amorphous nature, the CP was characterized using pair-distribution function (PDF) analysis and solid-state NMR (ssNMR), which showed that Zr–Phytate consists of octahedral ZrO_6_ centers which are bridged and cross-linked by phosphate groups from phytic acid. Beyond this, density functional theory (DFT) calculations revealed that the high selectivity toward Pb^2+^ is attributed to an exceptionally strong bond formed with PO_4_ groups. Further, PDF combined with DFT were employed to elucidate the likely Pb^2+^ binding mechanism. We also showed that Pb-loaded Zr–Phytate can be readily regenerated using 1 M HCl, maintaining its local structure and showing no drop in adsorption capacity or leaching over multiple adsorption/desorption cycles.

**Figure 1:**
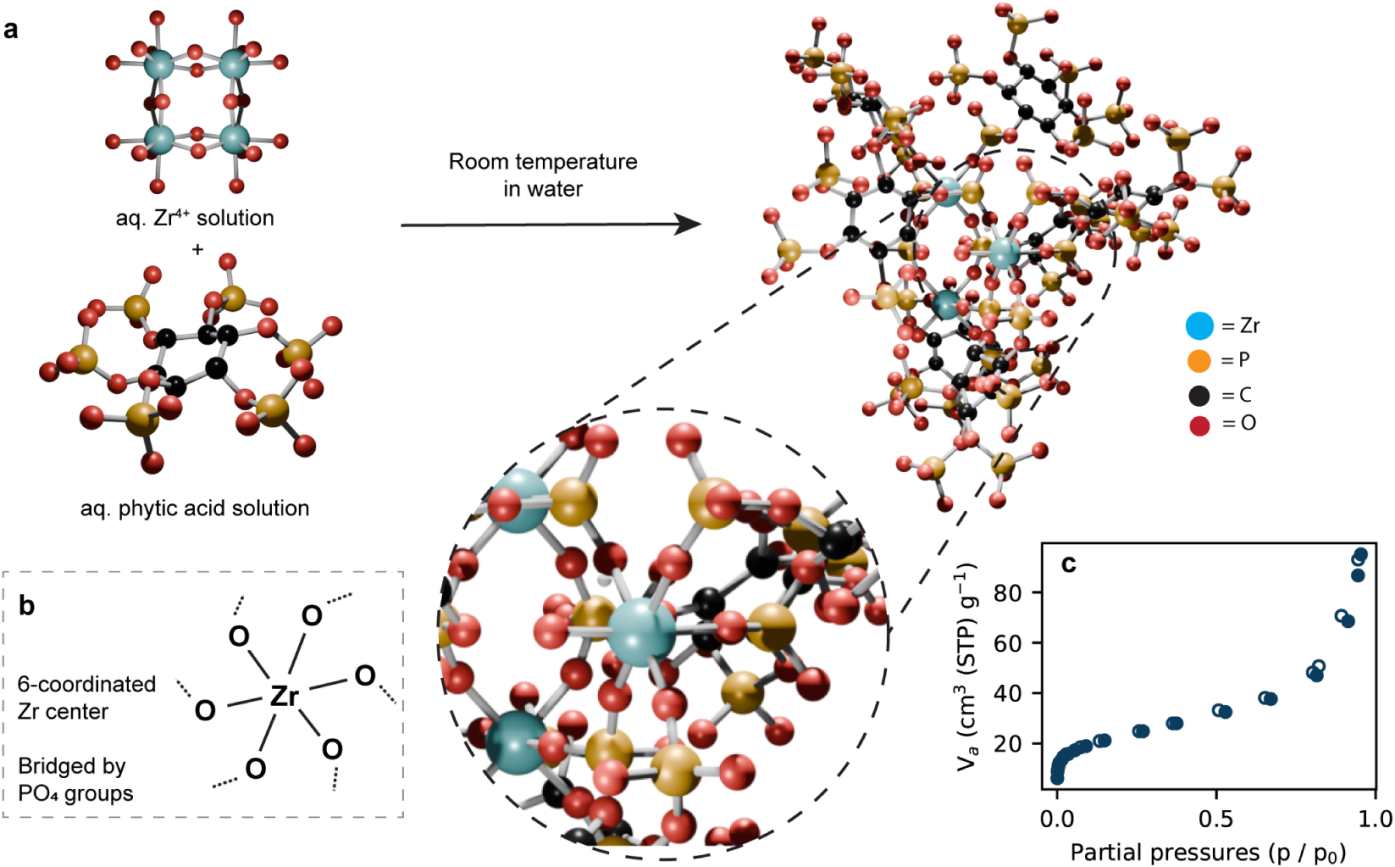
(a) Schematic representation of Zr–Phytate synthesis and structure, (b) Zr coordination environment in Zr–Phytate, and (c) N_2_ adsorption/desorption (solid/empty circles) isotherm of as-synthesized Zr–Phytate.

## Results and Discussion

### Characterization

The Zr–Phytate coordination polymer was formed by mixing an aqueous solution of Zr^4+^ salt and phytic acid solution together at room temperature, after which a white precipitate immediately forms (see Methods for details). A series of Zr–Phytate compounds (denoted as Zr–phytate-*X* where *X* = **1**, **2**, **3**, **4**) with increasing Zr/phytic acid ratios (from **1** to **4**) were first synthesized to identify the most effective ratio for Pb^2+^ removal. The Zr/phytic acid ratio was varied with the expectation that this would modify the number of available uncoordinated phosphate groups, which can bind Pb^2+^ and affect the performance. The results presented in Figure S1a-b indicate that Zr–Phytate-**1** outperformed the other adsorbents, matching our expectation that a lower amount of Zr in the structure would free up more adsorption sites. Based on these findings, Zr–Phytate-**1**, as shown in Figure 1, was selected as the best adsorbent, and all subsequent characterization was conducted using this material.

The mean Brunauer–Emmett–Teller (BET) surface area (*S*_BET_) calculated from N_2_ adsorption data of two independently synthesized batches of Zr–Phytate (Figures 1c and S3) was *S*_BET_ = 89(8) m^2^/g. The BET plots are shown in Figure S4. The isotherm shape, with the upturn at higher relative pressures, is indicative of powders containing mesopores that are either in the structure itself or formed between small particles. This is further supported by the pore size distribution plots (Figure S5), which show a broad range of mesopores (>2 nm). Powder X-ray diffraction of Zr–Phytate showed that the structure is amorphous (Figure S6b). The broad scan X-ray photoelectron spectrum (XPS, Figure S7a) contains characteristic peaks corresponding to Zr (3s, 3p, and 3d), P (2p), C (1s), and O (1s and KLL). The absence of a Cl 2p peak in the wide XPS spectrum of Zr–Phytate indicates that no residual precursor salt is present in the structure. Additionally, as shown in Figure S7b,c, the observed shift, relative to the precursor zirconium salt, to higher binding energies for both Zr 3d and P 2p peaks suggests a change in the electron density, which is indicative of a strong interaction between Zr and phytic acid. The broadening of the P 2p peak observed after the reaction of phytic acid with Zr^4+^ indicates that the phosphate groups within the polymer experience a range of chemical environments. This implies the presence of phosphate groups with varying numbers of Zr–O bonds. Elemental and thermogravimetric analyses (EA and TGA) were used to quantify the amount of Zr and phytic acid in the structure (details are provided in the Supporting Information). EA provided the amount of carbon, allowing for a straightforward calculation of the phytic acid weight percentage. The measured carbon content, 7.25(9)%, corresponds to 66.4(8)% of phytic acid. The Zr wt % was determined using TGA (Figure S8a), calculated from the residue mass of ZrP_2_O_7_ ^53^ which was found to be the decomposition product via PXRD (Figure S8b). The calculated Zr content was 23.4(2) wt %, and we estimate the overall phytic acid/Zr mass ratio to be 2.84(6). The corresponding molar phytic acid:Zr ratio is ∼0.4. Given that phytic acid contains six phosphate groups per molecule, the PO_4_:Zr ratio is ∼2.4. This is significantly higher than what is expected in Zr-phosphate glasses (Zr­(HPO_4_)_2_) where the PO_4_:Zr ratio is ∼2.

It was hypothesized that the greater number of phosphate groups in Zr–Phytate would enable higher metal ion uptake compared to Zr­(HPO_4_)_2_. So, the cation exchange capacity of Zr–Phytate was measured via titration with NaOH (Figure S2). The calculated exchange capacity, 11.1 milliequivalents (meq) of Na^+^ per gram of Zr–Phytate, significantly exceeds the reported capacities of Zr­(HPO_4_)_2_, which are in the range of 5.81–6.71 meq per gram.^32^ While we cannot necessarily correlate the calculated exchange capacity with the number of metal-bound versus unbound P–O groups in Zr–Phytate (since phosphate groups can coordinate metals even when protonated as P–OH),^36^ these results do indicate that Zr–Phytate likely has a higher density of available adsorption sites for metal ions than conventional Zr­(HPO_4_)_2_, supporting our hypothesis.

The Fourier-transform infrared (FTIR) spectrum of Zr–Phytate (Figure S6a) reveals a peak at 1393 cm^–1^, assigned to CH_2_ bending vibrations, indicative of the cyclohexane ring within the Zr–Phytate matrix. Peaks at 496 cm^–1^ and 683 cm^–1^ correspond to Zr–O stretching, confirming Zr integration into the CP structure. A broad, intense peak from 908 to 1150 cm^–1^ is characteristic of P=O, P–O, and C–O stretching vibrations, while peaks at 865 cm^–1^ and 807 cm^–1^ are related to Zr–O–P and P–O–C symmetric stretching. The peak at 1632 cm^–1^ suggests the presence of water, and the broad peak from 2300 to 3400 cm^–1^ indicates possible hydrogen bonding from hydroxyl groups (P–OH).^54–56^ These findings confirm the successful formation of Zr–Phytate.

Due to the amorphous nature of Zr–Phytate, PDF analysis was used to investigate the local structure of the CP. Figure 2a shows the PDF of Zr–Phytate and the phytate sodium salt. The first peak around 1.5 Å in Zr–Phytate aligns with the first peak in phytate and matches the expected P–O bond length in the phosphate group.^33^ Additionally, there are sharp peaks in Zr–Phytate that are absent in pristine phytic acid. These likely stem from the formation of Zr-phosphate clusters, which are also found in Zr-phosphate (Zr­(HPO_4_)_2_). To verify this, we fitted the PDF of Zr–Phytate with structural models of the most common polymorphs, α- and τ-Zr­(HPO_4_)_2_, of which the latter fits relatively well (Figure S10). The peaks centered around 2 and 3.5 Å in the PDF of Zr–Phytate are, however, not very well fitted, as can be seen in the difference curve in Figure S10. This is mainly due to the contribution of phytic acid to the PDF, which is absent in τ-Zr­(HPO_4_)_2_. To isolate the features of the Zr-phosphate interactions, the differential PDF (dPDF) was calculated by subtracting the PDF of the phytate salt from that of Zr–Phytate (red curve in Figure 2a). We then extracted a set of discrete Zr-phosphate clusters with one to three Zr atoms and varying ratios of Zr:PO_4_ groups from a 2 × 2 × 2 supercell of the reported τ-Zr­(HPO_4_)_2_ structure. More details are provided in the Supporting Information. The PDF of each cluster was simulated by using DiffPy-CMI and fitted to the dPDF by using a linear combination of the cluster PDFs. The results are shown in Figure S11, and we can see that the peaks centered around 2 and 3.5 Å have much better fits. The main contribution of the linear combination comes from two clusters with compositions Zr_3_(PO_4_)_4_ and Zr_2_(PO_4_)_11_ (see Figure 2d). Figure 2e shows a two-phase model fit of those two structures to the dPDF. The refinement results are shown in Table S2. This indicates that Zr in Zr–Phytate is similarly coordinated as in τ–Zr­(HPO_4_)_2_, i.e., it consists of 6-connected octahedral ZrO_6_ centers. To support this, we also looked to the MOF literature. To date, while there are few cases where single crystals of Zr-phosphonate MOFs suitable for single crystal X-ray diffraction have been obtained, we find the reported structures are also comprised of 6-coordinate ZrO_6_ octahedra.^42^ More importantly, the position of the Zr–P peak at around 3.5 Å strongly indicates that the phosphate groups are forming bridging rather than chelating bonds with Zr. Due to the much smaller Zr–O–P bond angle, chelating binding modes would lead to significantly shorter Zr–P peaks than observed in the dPDF. Most previously reported studies on Zr–Phytate compounds assume, although lacking any experimental evidence, chelating binding modes and 4-coordinated Zr centers.^48,57^ This is the first example of a structural investigation into Zr–Phytate.

**Figure 2:**
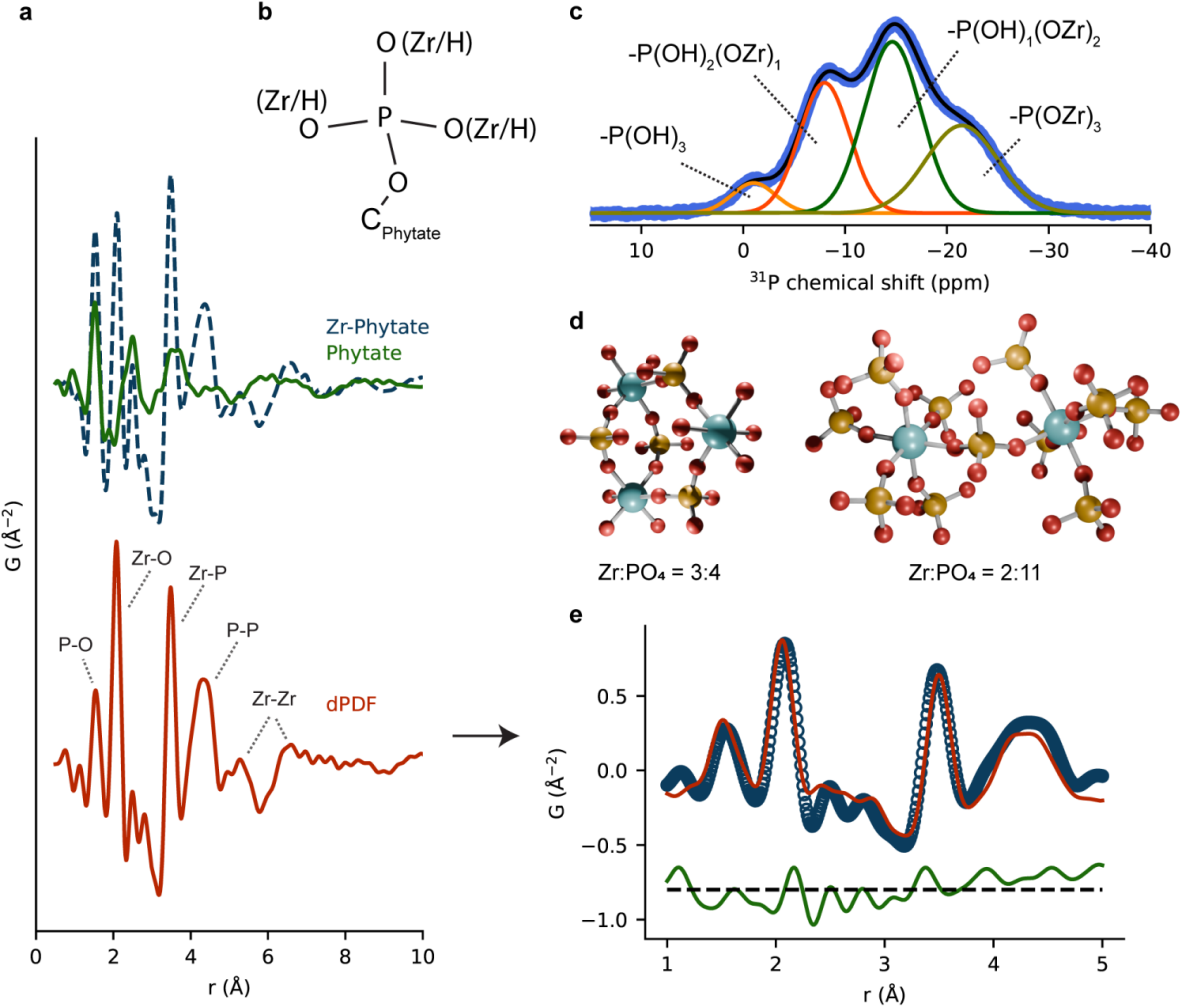
(a) PDF of Zr–Phytate (blue), phytate (green), and the differential PDF (red); (b) scheme of the phosphate group appended to the hexane backbone of phytic acid, with three available oxygen groups which may be protonated, unprotonated, or bound to Zr^4+^; (c) ^31^P NMR of Zr–Phytate including deconvolution of the phosphate peaks; (d) scheme showing the discrete structural models that were extracted from τ-Zr­(HPO_4_)_2_ and used for PDF fitting; the coloring code is as follows: Zr (light blue), O (red), and P (orange); (e) two-phase model fit of the dPDF shown in (a) using the two discrete structural Zr-PO_4_ clusters shown in (d). The fitted parameters are listed in Table S2.

Due to the limited real-space resolution of the PDFs, we use ^31^P NMR to get an understanding of the range of chemical environments in the phosphate groups of Zr–Phytate. The ^31^P spectrum of crystalline, ordered layered α-Zr­(HPO_4_)_2_ typically features a single sharp peak centered around −20 ppm^58^ attributed to phosphate groups with three P–O–Zr bonds. The same would be expected for a crystalline τ-Zr­(HPO_4_)_2_ structure. The broad signal measured for Zr-Phytate (Figure 2c) clearly indicates the presence of multiple phosphate environments. Figure 2c shows a broad signal featuring 4 shoulders, which can be fitted well with 4 Gaussians, centered at −1.02 (6%), −7.98 (27), −14.64 (40%), and −21.47 (27%) ppm. Each corresponds to a distinct chemical environment in PO_4_ groups as observed in Zr-phosphate structures.^59^ The formation of P–O–Zr bonds creates a shift toward lower fields,^60^ and the peaks at −7.98, −14.64, and −21.47 ppm can be attributed to phosphate groups with one, two, and three Zr–O–P bonds, as shown in Figure 2c. The peak at −1.02 ppm is likely coming from free phosphate groups. PDF analysis revealed that the local structure of Zr–Phytate resembles that of τ-Zr­(HPO_4_)_2_, whereas ^31^P NMR shows that Zr–Phytate contains a range of Zr-phosphate binding modes, including a substantial number of free phosphate groups that are not bound to Zr.

### Adsorption Experiments

To evaluate the adsorption capacity and efficiency of Zr–Phytate for Pb^2+^ capture, we measured two important performance metrics: Pb^2+^ adsorption isotherms and kinetics in environments with both high and low Pb^2+^ concentrations, with the latter being particularly important for water purification applications. Adsorption isotherms were measured using Pb^2+^ solutions with starting concentrations ranging from 5 to 100 mg/L and equilibrium concentrations (*c*_e_) spanning from 0.005 to ∼55 mg/L. Moreover, the adsorption behavior of Zr–Phytate was compared to that of Zr-phosphate, referred to as Zr­(HPO_4_)_2_, to investigate the advantages of using phytic acid as a cross-linking agent compared to phosphoric acid. Zr­(HPO_4_)_2_ was synthesized under conditions similar to those used for the synthesis of Zr–Phytate (see Supporting Information for more details). As shown in Figure 3a, Zr–Phytate is capable of adsorbing much larger quantities of Pb^2+^ at low concentrations, with a maximum experimentally determined capacity of 427(10) mg/g compared to 181(7) mg/g for Zr­(HPO_4_)_2_.

**Figure 3:**
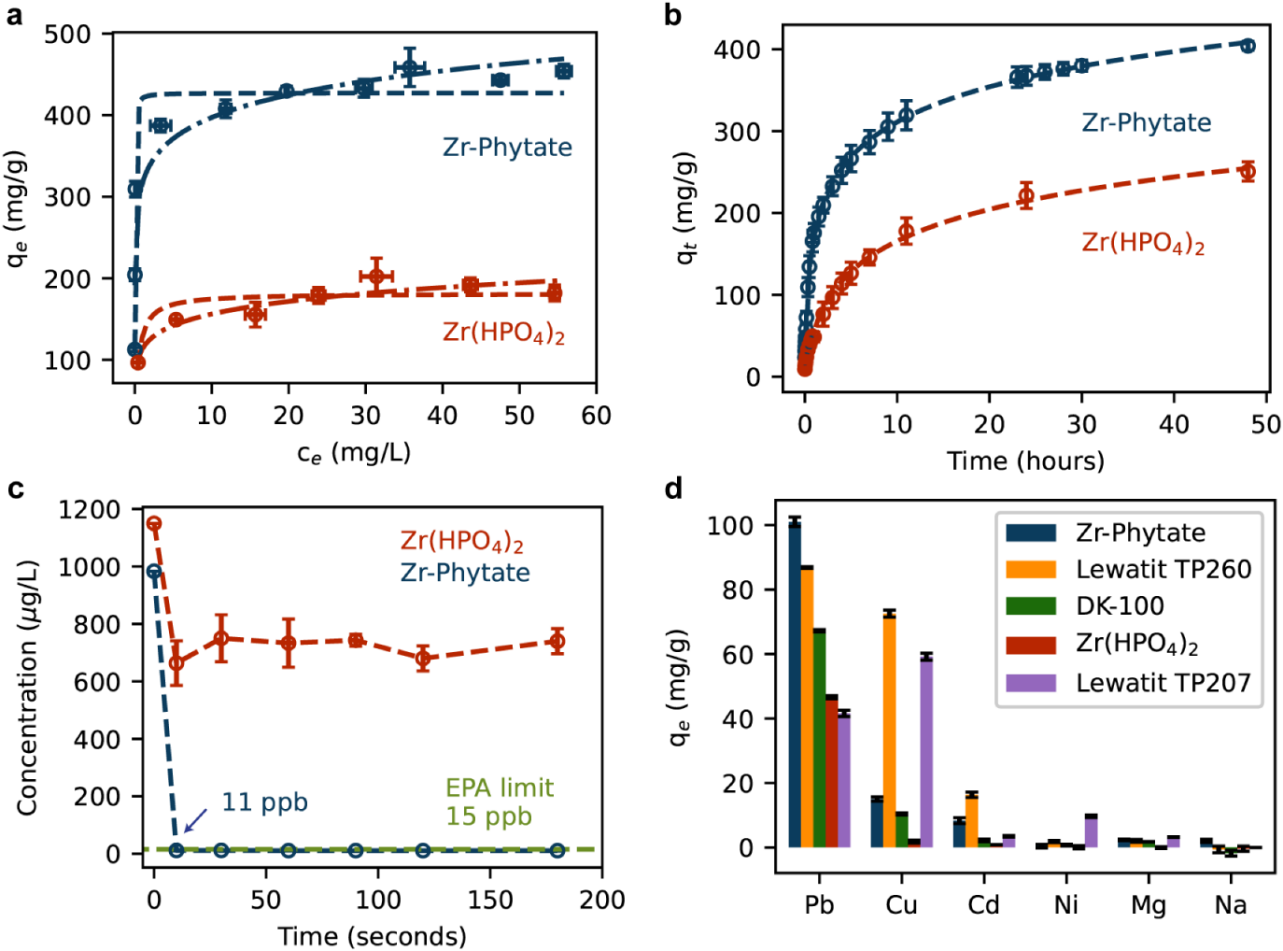
(a) Isotherms of Zr-Phytate (blue) and Zr­(HPO_4_)_2_ (red) using Pb^2+^ concentration: 10–100 mg/L (Zr­(HPO_4_)_2_), 5–100 mg/L (Zr–Phytate) and an adsorbent dosage: 0.1 mg/mL, time: 48 h. The experimental data are plotted in ″circles″, the Langmuir fit in a ″dashed″ line, and the Freundlich fit in “dashed-dotted” line. (b) Kinetic study of Zr–Phytate compared to Zr­(HPO_4_)_2_. The dashed line is the fitted Elovich adsorption model. Initial Pb^2+^ concentration 50 mg/L; adsorbent dosage: 0.5 mg/mL. (c) Kinetic study of Zr–Phytate and Zr­(HPO_4_)_2_ at low concentrations. Initial Pb^2+^ concentration: 1 mg/L and adsorbent dosage: 0.5 mg/mL. (d) Selectivity experiment with initial metal ion concentration: 100 mg/L, adsorbent dosage: 1 mg/mL, and time: 48 h. The error bars represent standard deviations from triplicate experiments.

Next, we fitted two adsorption models (Langmuir and Freundlich) to the data, which provided some insight into the characteristics of Zr–Phytate as an adsorbent for heavy metals. As can be seen from the fitted parameters in Table 1, the Langmuir model fits better to the Zr–Phytate isotherm. The lower *R* ^2^ of the Freundlich fit (0.9052) results mainly from a worse fit of the low-concentration points (*c*_e_ below 1 ppm). The Langmuir model offers a better fit, resulting in an *R* ^2^ of 0.9469. The Langmuir model assumes a surface with homogeneous adsorption sites, i.e., uniform adsorption sites with equal binding energies, whereas the Freundlich model typically fits better to adsorbents with various adsorption sites.^61^ Interestingly, the situation is reversed for Zr­(HPO_4_)_2_, where the Freundlich model fits better (*R* ^2^ = 0.8964) than the Langmuir model (*R* ^2^ = 0.7958). Although the adsorption sites are expected to be chemically similar in both cases, Zr–Phytate is more porous than Zr­(HPO_4_)_2_ (see Figure S9), which could result in better and more uniform accessibility of adsorption sites. Importantly, the adsorption behavior at low concentrations is more pertinent given that lead is typically found well below 1 ppm. At trace concentrations, the Langmuir model reduces to a linear form and follows Henry’s law.^62,63^ In the Henry regime, the number of adsorption sites is considered to be in excess compared with the solutes. Therefore, the product *K*_L_ × *q*_max_ (L/g) is a good predictor of lead affinity under environmentally relevant conditions. The adsorption isotherm of Zr–Phytate follows the Langmuir model well, and the model indicates that the material has a strong affinity to Pb^2+^ at low concentrations with *K*_L_ *q*_max_ = 2.9(6) × 10^4^ compared to 4(2) × 10^2^ for Zr­(HPO_4_)_2_. Importantly, these values are higher than those we have calculated for other adsorbents reported in the literature (Table S1). On the other hand, the Freundlich model does not follow Henry’s law at low concentrations. So, instead we used the fitted Freundlich parameters to calculate the capacities at *c*_e_ = 500 ppb Pb^2+^, estimating the performance at low concentrations. Comparing *q*_e_ at 500 ppb using the Freundlich equation shows a similar trend, with *q*_*e*_500*ppb*_ equal to 297(21) and 104(23) for Zr–Phytate and Zr­(HPO_4_)_2_, respectively. We suspect that the overall higher Pb^2+^ adsorption capacity and the much steeper adsorption isotherm of Zr–Phytate at low Pb^2+^ concentrations are due to the higher availability of unsaturated P–O groups compared to Zr­(HPO_4_)_2_.

**Table 1:**
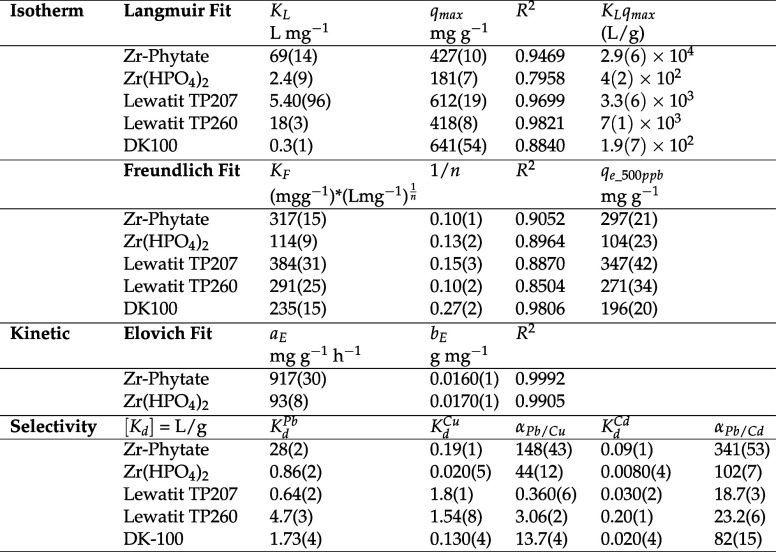
Fitting Parameters of Adsorption Models (Kinetics and Isotherms) and Distribution Coefficient Calculated from the Selectivity Experiments in Figures 3b,d and S12a–c ^a^

a The uncertainties are calculated from the squared diagonal matrix elements of the covariance matrix used for the nonlinear regression models. The uncertainty for the product *K*_L_ *× q*_max_ was calculated by propagating the standard errors of both terms through the multiplication. For the predicted Pb^2+^ uptake at 500 ppb (*q_e_500ppb_*), the given uncertainty is the 80% confidence interval determined by using the delta method. Details are provided in the Method Section.

Figure 3b shows the Pb^2+^ uptake of both materials plotted as a function of time. We fitted various kinetic adsorption models to the data, including the pseudo-first-order (*R* ^2^ = 0.9147), the pseudo-second-order (*R* ^2^ = 0.9656), the intraparticle diffusion model (*R* ^2^ = 0.8830), and the Elovich adsorption model (*R* ^2^ = 0.9992). Among those models, the Elovich model (Figure 3b and Table 1), typically used to describe chemisorption processes on adsorbents^64^ fits the data much better than all three other models. The results of the other kinetic models are shown in the Supporting Information (Figure S13). Comparing the kinetic data of Zr–Phytate to that of Zr­(HPO_4_)_2_, it is shown that the latter has much lower diffusion rates than Zr–Phytate. The Elovich parameter *a*_E_, i.e., the initial adsorption rate (Table 1), is roughly ten times higher in Zr-Phytate (917(30) mg g^–1^ h^–1^) than in Zr­(HPO_4_)_2_ (93(8) mg g^–1^ h^–1^). Further, the removal efficiency of Zr­(HPO_4_)_2_ and Zr–Phytate at low concentration (Figure 3c) shows that only the latter effectively removes Pb^2+^ below limits defined by the Environmental Protection Agency (EPA).^65^ Given that lead concentration in water is generally low,^66^ this data indicates that Zr­(HPO_4_)_2_ is less promising. This underscores the advantage of employing phytic acid over H_3_PO_4_, as its higher density of free phosphate groups offers more coordination sites for metal ions, thereby enhancing the Pb^2+^ uptake.

Given the high performance, we also tested Zr–Phytate against three commercial resins used as benchmarks for Pb^2+^ removal. For this, we selected Lewatit TP207 (Lewatit MonoPlus TP207)^67^ and Lewatit TP260 (Lewatit MonoPlus TP260)^68^ because they are specifically designed for heavy metal removal and reportedly have high selectivity toward Pb^2+^ over commonly encountered metals. We also included DK-100 (Diaiontm WK100)^69^ which is suitable for a wider application range and is reported to have high regeneration efficiency. All selected resins are weakly acidic cation exchange resins and offer varying heavy metal binding functionality. For example, Lewatit TP260 contains chelating aminomethylphosphonic acid groups^68^ which are expected to be chemically similar to the functional groups in Zr–Phytate. Lewatit TP207 contains iminodiacetic acid groups, which are also well-known to chelate heavy metals, and DK-100 primarily features surface carboxylic acid groups. We measured Pb^2+^ adsorption isotherms in lead-only solutions for all three resins (Figure S12) and then fitted the data using Langmuir and Freundlich models. The results of the fits are listed in Table 1. Importantly, the resins have high maximum Pb^2+^ uptake capacities, with TP207 and DK-100 each adsorbing ∼600 mg/g and TP260 ∼400 mg/g. However, the adsorption behavior at low concentrations is more application-relevant. Thus, the products (*K*_L_ *q*_max_) are listed in Table 1. It is clear that Zr–Phytate, with *K*_L_ *q*_max_ = 2.9(6) × 10^4^ has a stronger affinity to Pb^2+^ than Zr­(HPO_4_)_2_ and the tested resins (1.9(7) × 10^2^, 7(1) × 10^3^, and 3.3(6) × 10^3^ L/g for DK100, TP260, and TP207, respectively). Using the product term, Zr–Phytate also exceeds other top-performing materials listed in the literature (Table S1); we find that *K*_L_ *q*_max_ is orders of magnitude higher than that of all of these previously reported materials. For instance, a Zr-MOF with the highest reported *q*_max_ of 1309 mg/g only has a *K*_L_ *q*_max_ of 5 L/g.^31^ The highest product term in Table S1 is *K*_L_ *q*_max_ = 588 for a Zn-BDC.^26^ However, this MOF is unstable in water and thus not suitable for commercial use. The larger product term of Zr–Phytate, which is two orders of magnitude higher than other top performing materials (Table S1) and one order of magnitude higher than several commercial ion-exchange resins tested in this work, underscores its high affinity for lead, which is needed for an adsorbent to be effective in highly competitive, environmentally relevant conditions.

Given the higher affinity of Zr–Phytate at low concentrations when tested in solutions containing only lead, we next tested the Pb^2+^ uptake in competitive environments (Figure 3d with initial and final equilibrium concentrations in Table S4). For this, we evaluated the selectivity of Zr–Phytate, Zr­(HPO_4_)_2_, and the commercial resins toward Pb^2+^ over other interfering toxic (Cu, Ni, Cd) and nontoxic metal (Mg, Na) ions with a starting concentration of 100 mg/L for all ions. Importantly, under these conditions, Zr–Phytate exhibits far superior selectivity toward lead, with the highest Pb^2+^ uptake of all materials tested. Lewatit TP207 exhibits a higher selectivity toward Cu^2+^ over Pb^2+^ (Figure 3d) and also readily takes up other cationic species like Cd^2+^, Ni^2+^, and Mg^2+^. In contrast, DK-100, Lewatit TP260, Zr­(HPO_4_)_2_, and Zr–Phytate show greater selectivity for Pb^2+^ over the other tested cations, although TP260 also has a substantial uptake of Cu^2+^. To have a more quantitative assessment, we calculate the selectivity factor α as the ratio of *K*_d_^Pb^ over the *K*_d_ value of a competing ion from the same experiment, allowing a comparison of each adsorbent’s affinity toward the different metals (Table 1). We note that α could be calculated only for Pb:Cu and Pb:Cd because the uptake of the other metals is negligible in most cases, which makes the selectivity calculations and thus a comparison between adsorbents erroneous. The selectivity experiment shows that phosphate groups are highly selective adsorption sites for Pb^2+^. For example, although the overall Pb^2+^ uptake of Zr­(HPO_4_)_2_ in competitive conditions is lower than that of DK-100, the selectivity of Zr­(HPO_4_)_2_ for Pb^2+^ over Cu^2+^ and Cd^2+^ is greater than all three resins. The selectivity factors (α) of Zr­(HPO_4_)_2_ for Pb^2+^ over Cu^2+^ and Cd^2+^ are 44(12) and 102(7), respectively, compared to 13.7(4) and 82(15) for DK-100. The higher selectivity of Zr­(HPO_4_)_2_ toward Pb^2+^ is likely due to the stronger affinity of Pb^2+^ to phosphate groups than carboxylic acid groups present in DK-100. This is further supported by the performance of Zr–Phytate in a competing environment with α values of 148(43) for Pb^2+^ over Cu^2+^ and 341(53) for Pb^2+^ over Cd^2+^. The superior uptake of Zr–Phytate (Figure 3d) stems from its higher selectivity toward Pb^2+^ over Cu^2+^ and Cd^2+^ when compared to all three commercial resins and Zr­(HPO_4_)_2_. This indicates that Zr–Phytate has a significantly higher density of free phosphate groups available to bind Pb^2+^ compared to Zr­(HPO_4_)_2_ and that phosphate groups bind Pb^2+^ exceptionally well compared to the other chemical functionalities present in the resins.

Next, we assessed the performance of Zr–Phytate under more challenging conditions by using a low Pb^2+^ concentration of 1 mg/L while progressively increasing the concentrations of competing ions to 10 and 100 mg/L (Figure 4 and initial and final equilibrium concentrations are listed in Tables S5–S7). This resulted in Pb^2+^ to competing ion ratios of 1:1, 1:10, and 1:100, respectively. These ratios refer to single-element comparisons. If we sum all competing metal ions present in the solution and compare them to the Pb^2+^ concentration, we get ratios of 1:6, 1:60, and 1:600. This experiment is important because an ideal adsorbent must maintain high performance at low contaminant concentrations and in the presence of high concentrations of competing metal ions. In the 1:1 experiment shown in Figure 4a, Zr–Phytate removes almost all Pb^2+^, Cu^2+^, and Cd^2+^ and has a significant uptake of Ni^2+^, Ca^2+^, and Mg^2+^ as well. While a similar trend is observed for the two Lewatit resins (TP260 and TP207), DK-100 has a notably lower removal efficiency for all tested metal ions. At a ratio of 1:1, we can already observe that among the tested materials, Zr–Phytate offers the highest Pb^2+^ removal efficiency (>99%). On the contrary, the tested resins are slightly lower with Pb^2+^ uptakes ranging from 83(2) to 88(4)%. Importantly, as the concentration ratio of Pb^2+^ to interfering ions is increased from 1 to 10 or 1 to 100 ppm, the differences in selectivity become much more apparent. Figure 4b,c reveals only a small drop in the Pb^2+^ removal efficiency for Zr–Phytate, while all resins already have a much more pronounced drop in performance at a 1:10 ratio. After the concentration of competing ions is increased by a factor of 100, the Pb^2+^ uptake only drops slightly to ∼92% for Zr–Phytate, whereas it drops to less than 45% for all commercial resins. These results highlight the superior selectivity of Zr–Phytate for Pb^2+^ removal, even under highly competitive conditions, where commercial resins proved to be ineffective.

**Figure 4:**
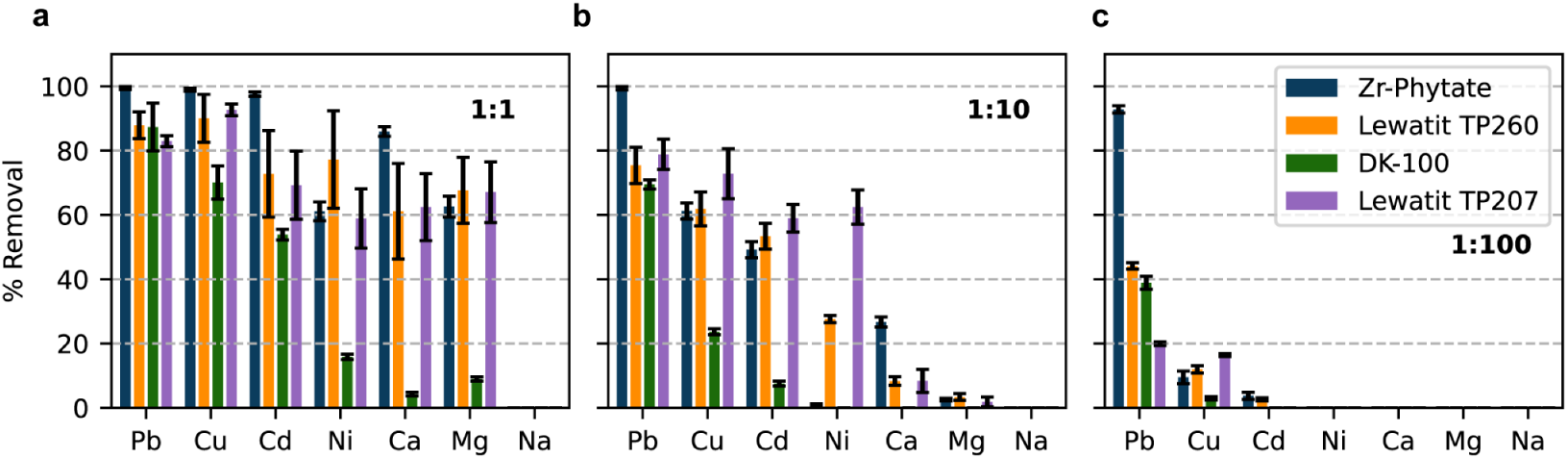
Selectivity experiments with various ratios of Pb^2+^:*I* where *I* refers to interfering ions. Each solution contained a mixture of all elements. The initial Pb^2+^ concentration was *c*_0_ = 1 mg/L in all cases, whereas the concentrations of each interfering ion were 1 (a), 10 (b), and 100 (c) mg/L. The adsorption time was 48 h, and the adsorbent dosage was 0.25 mg/mL. The error bars represent standard deviations from triplicate experiments.

While the high Pb^2+^ removal efficiency in the presence of excess amounts of interfering metal ions in Figure 4 is encouraging, the question remains as to how well Zr–Phytate can remove Pb^2+^ ions in a fixed adsorbent bed under flow. The removal efficiencies in Figure 4 are determined under equilibrium conditions, whereas adsorption in a fixed bed operates under nonequilibrium conditions^70^ where kinetic parameters significantly influence the removal efficiency.^70,71^ We therefore flowed 40 mL of solution containing 1 mg/L Pb^2+^ and six other competing ions in a 1:1 and then 1:00 ratio through the column (1 mL/min) and subsequently determined the metal ion removal efficiencies via ICP. The experimental setup is shown in Figure S17. The results in Figure 5 show that the selectivity toward Pb^2+^ remains high under both conditions. For the 1:1 case, the Pb^2+^ uptake is close to 100%, whereas the percentage removal of the other cations is substantially lower than that observed in the equilibrium experiments (Figure 4a). This enhanced selectivity under flow condition can be attributed to two complementary factors. First, kinetic effects may favor Pb^2+^ adsorption, as lead ions may exhibit faster adsorption compared to competing cations, allowing preferential uptake during the limited contact time in the flow system. Second, the stronger, competitive adsorption of lead decreases the uptake of the other ions under flow conditions by blocking the ions having significantly lower affinities to the adsorbent surface.^70^

**Figure 5:**
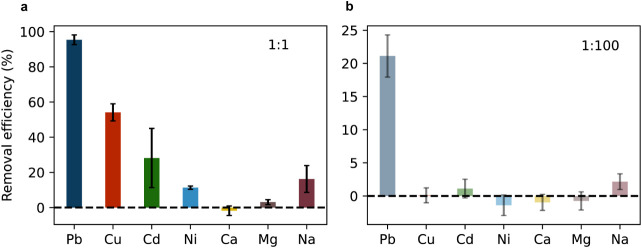
Fixed bed flow-through adsorption experiment with Zr–Phytate. The metal cations in solutions are listed on the *x*-axes. The concentration of Pb^2+^ was 1 mg/L, and that of each interfering metal ion was (a) 1 or (b) 100 mg/L. The removal efficiency was calculated from the adsorbed metal ions of 40 mL of metal ion solution flown through a fixed bed with a flow rate of 1 mL/min. The error bars represent standard deviations from triplicate experiments.

For the 1:100 case (Figure 5b), the Pb^2+^ removal efficiency dropped compared to that in the 1:1 case, while the selectivity remained high. This reduction is likely due to mass transport limitations of Pb^2+^ ions caused by the increased ionic strength of the solution.^72,73^ Additional effects, such as electric screening of adsorption sites and pore crowding, can further impact adsorption efficiency under high ionic strength conditions.^74^ Importantly, these effects diminish the removal efficiency of the other cations as well, and the high selectivity toward Pb^2+^ is maintained even in the presence of excess amounts of competing ions. While these flow-through results demonstrate that Zr–Phytate maintains promising selectivity under nonequilibrium conditions, commercial application will require developing and optimizing protocols to structure the fine powder into beads or pellets that provide optimal mass transport while maintaining high selectivity toward lead.

To better understand the Pb^2+^ binding mechanism in Zr–Phytate, we conducted ^31^PNMR on powders after Pb^2+^ adsorption. In Figure S14, the spectrum of the as-synthesized Zr–Phytate is compared to that of the sample loaded with 8 and 15 wt % of lead. The ^31^PNMR spectra after Pb^2+^ adsorption do not change significantly. The signal is still shaped by 4 peaks, and no new shoulder that could be assigned to a P–O–Pb bond appears. However, we note that the peak centered around −1 ppm, previously assigned to free and protonated P–O–H bonds, becomes gradually broader (Table S8). The peak width changes from 5.9 to 7.5 and 8.9 from the lead-free samples to the 8 and 15% lead-loaded samples, respectively. Second, all four peak positions shift gradually toward lower chemical shifts. This indicates that the Pb–O–P bonding likely has less of a covalent character than the Zr–O–P bonding, as Pb^2+^ incorporation does not appear as a strong new peak, but rather influences the broadness of the peaks and causes slight peak shifts in the ^31^PNMR spectra. In lead phosphate glasses, the Pb–O–P bonding is mostly characterized as ionic, although it can become more covalent in character at very high Pb^2+^ concentrations.^75^ This indicates that the Pb^2+^ adsorption mechanism is best described as an ion exchange mechanism in Zr–Phytate.

To further support this, we computed charges on the phosphate-oxygen groups in a protonated Zr­(H_2_O)_2_(H_2_PO_4_)_4_ cluster and a Zr­(H_2_O)_2_(H_2_PO_4_)_2_Pb­(HPO_4_)_2_ cluster. The structures were geometry-optimized using DFT calculations, and the charges on the atoms were computed via MBIS analysis (see Methods for details).^76^ The clusters are depicted in Figure S15, and the charges are listed in Table S9. We note that the charges on the phosphate-oxygens when protonated and when bound to lead are almost unchanged, showing that in both cases, the electron density is strongly localized on the O-groups. There is also a small decrease in phosphorus charges after binding Pb^2+^, which can explain the slight shift of the peaks in the ^31^PNMR spectra.

Given that Pb^2+^ ions are much larger than Cu^2+^, Ni^2+^, or Ca^2+^ cations, they are comparably much more polarizable. Thus, as shown in many literature examples, Pb–O bonds have a non-negligible covalent character.^77^ This higher covalency is likely partly responsible for the high selectivity toward Pb^2+^ compared to the other cations tested in Figure 4. For instance, looking at tabulated metal-phosphate solubility constants, we find that *K*_S_ ≈ 10^–80^ for Pb_5_(PO_4_)_3_Cl^78^ whereas for transition metal-phosphates, like Cd_3_(PO_4_)_2_, Cu_3_(PO_4_)_2_, and Ni_3_(PO_4_)_2_, they are in the order of 10^–37^–10^–32^ and for even less polarizable alkaline earth metal-phosphates like Mg_3_(PO_4_)_2_ and Ca_3_(PO_4_)_2_, they are 1.04 × 10^–24^ and 2.07 × 10^–33^, respectively.^79^ The solubility of Li_3_PO_4_, a complex with an alkali metal, i.e., a very hard cation, has *K*_S_ = 2.37 × 10^–11^,^79^ which is orders of magnitude higher than those of alkaline earth and transition metals. This indicates that phosphate-based adsorbents are much more effective in immobilizing post-transition metals such as Pb^2+^ cations compared to other metal ions commonly present in water.

Next, we look for structural changes in Zr–phytate after Pb^2+^ adsorption by using PDF analysis. For this, we compared the PDF of the sample containing 15 wt % Pb to the as-synthesized powder. Figure 6a shows the PDFs of the pristine CP and Pb-adsorbed CP plotted on top of each other, and the difference curve is plotted below in red. Despite the relatively high Pb^2+^ loading, there is minimal change in the local structure except in the range between 2.0 and 2.5 Å, where both Zr–O and Pb–O bonds are located. The average Pb–O bond length in glasses is ∼2.3 Å,^80^ which matches the range in the PDF where new features appear in the difference curve (highlighted in yellow). This also matches Pb–O distances in the DFT-optimized structural models, which are depicted in Figure 6b. We considered two possible binding modes: a monodentate binding mode with a phosphate group bound to Pb^2+^ through one of its oxygen atoms and a bidentate/chelating binding mode with a phosphate group bound to Pb^2+^ through two of its oxygen atoms. To highlight the Pb–O bond distances in the models, protons and water ligands, which were considered for charge balance and to account for solvent effects, are omitted. The complete structural models are shown in Figure S15. The Pb–O distance from the DFT-optimized models matches well with the distance in the PDF where changes in the difference curve appear. Pb^2+^ is stable in a wide range of bonding environments, with common coordination numbers ranging from 6 to 12 ^81^ and Pb^2+^ likely also exists in a wide range of chemical environments in Zr–Phytate. Consequently, the local structure around the adsorbed Pb^2+^ cations is likely strongly disordered, which explains the relatively broad features appearing in the differential PDF in Figure 6.

**Figure 6:**
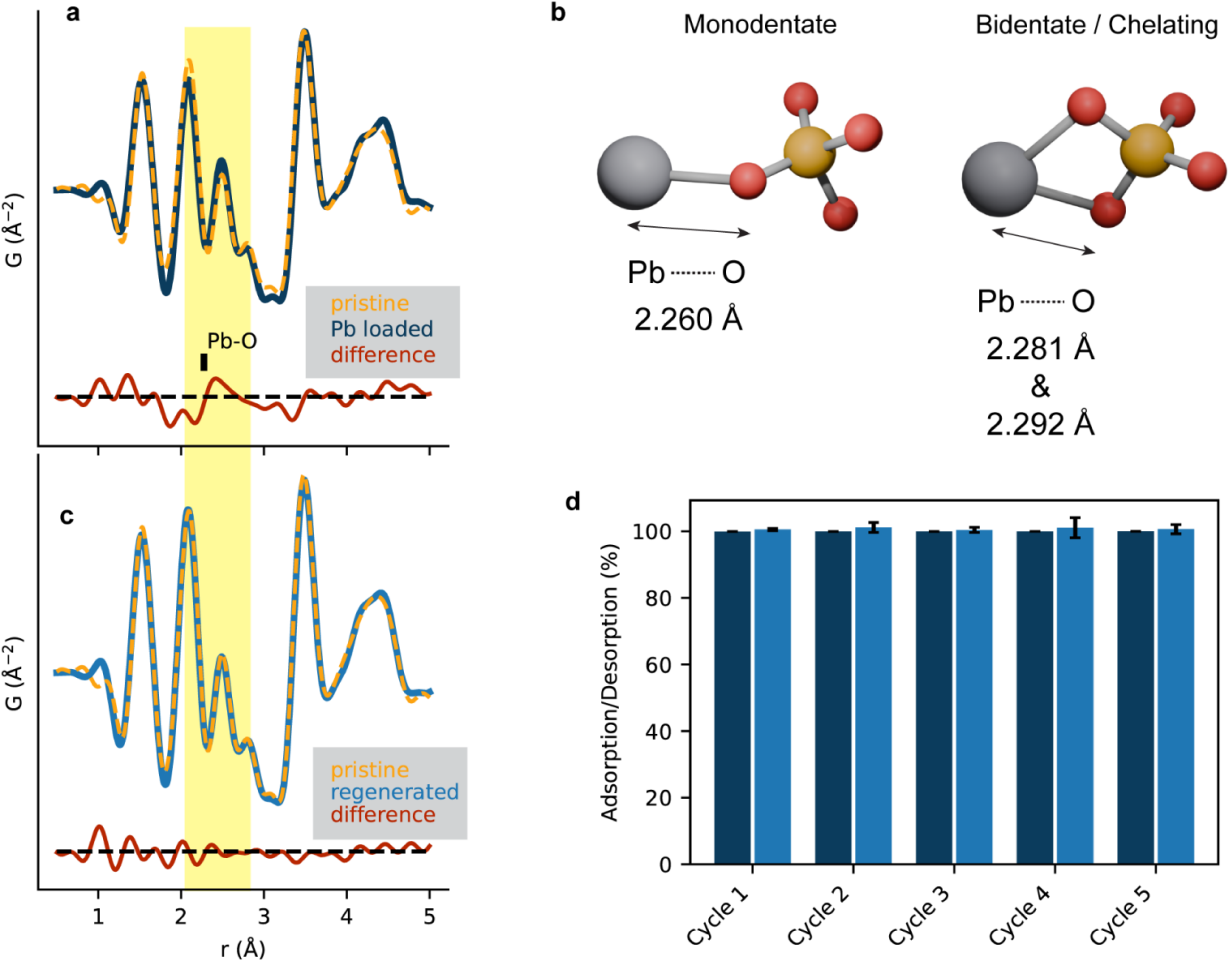
Regeneration experiment of Zr–Phytate showing the adsorption percentage (dark blue) and desorption percentage (light blue). Adsorption condition: Pb^2+^ concentration: 100 mg/L; adsorbent dosage: 5 mg/mL. Desorption condition: 1 M HCl solution. (a) PDFs of Pb-loaded Zr–Phytate (solid blue) and the as-synthesized Zr–Phytate (dotted yellow) with the difference curve below (solid red). The difference curves are calculated as difference = *G*_Pb‑loaded_ – scale × *G*_pristine_ and difference = *G*_regenerated_ – scale × *G*_pristine_, respectively, where the scale factor is chosen to minimize the difference curve. (b) Structural models of potential binding modes of Pb with phosphate groups. The structures are optimized using DFT calculations. For the sake of clarity, water ligands and protons are omitted. The full structures can be found in Figure S16. Color code: Pb (dark gray), P (orange), and O (red). (c) Comparison between the PDF of the regenerated CP (solid light blue) and the as-synthesized CP (dotted yellow) and the difference curve (solid red). (d) Consecutive adsorption–desorption cycles. Adsorption: dark-blue bars; desorption: light-blue bars. The error bars represent standard deviations from triplicate experiments.

To understand how phosphate group protonation affects Pb^2+^ binding and how phosphate ligands compare to common ion-exchange sites like acetate groups (DK100), we computed complex energies (Table S10 and Figure S16) and calculated Gibbs free energies (Δ*G*°) for various exchange reactions to identify thermodynamically favorable binding modes. We examined exchanges between monodentate acetate and phosphate ligands (*R*_1,2_), their chelating counterparts (*R*_3,4_), and cross-exchanges involving monodentate and bidentate configurations (*R*_5–7_), as detailed in Section 4.7 of the Supporting Information. The results demonstrate that the phosphate protonation state determines the preferred binding mode: H_2_PO_4_^–^ preferentially adopts monodentate coordination, while HPO_4_^2–^ favors chelating coordination (Table S10). Further, exchange energy calculations reveal that replacing chelating acetate ligands with chelating HPO_4_^2–^ is energetically favorable, as is the monodentate exchange of acetate with H_2_PO_4_^–^. Last, the DFT calculations indicate that preferred binding modes are strongly pH-dependent. Notably, H_2_PO_4_^–^ forms weaker complexes than HPO_4_^2–^, suggesting that at low pH, Pb^2+^ may be exchangeable with protons in Zr–Phytate systems (Table S10), which provides insight into potential regeneration.

Finally, we evaluated the regenerability of Zr–Phytate after exposure to Pb^2+^ solutions (Figure 6). Based on our findings from ^31^PNMR, PDF analysis, and DFT calculations, we think that the Pb^2+^ binding is weakly covalent, strongly ionic, and pH dependent. Further, Zr-phosphate structures are known to undergo ion-exchange readily and offer high proton conductivity^35,82^ promoting metal ion adsorption via replacement of protons. We suspect a similar mechanism occurs in Zr–Phytate where adsorbed Pb^2+^ could be replaced again by protons in sufficiently acidic solutions; thus, we tested the regenerability in 1 M HCl. Indeed, Pb^2+^ was effectively removed from Zr–Phytate (see the Methods section for more details). For the adsorption cycles, we used an adsorbent dosage of 5 mg/mL in a 100 ppm of Pb^2+^ solution. Importantly, over five successive adsorption cycles, we demonstrated that ∼100% of Pb^2+^ could be adsorbed and desorbed (Figure 6b) as confirmed by ICP-OES analysis. Moreover, no measurable quantity of Zr could be detected in the HCl solution.

To better assess the regenerability of the adsorbent, we evaluated the removal efficiency of Zr−Phytate before and after cycling experiments using a low adsorbent dosage (0.1 mg mL^−1^). This approach ensures that the removal efficiency remains well below 100%, allowing us to observe any decline in adsorption capacity—a feature often overlooked in experiments where the adsorbent is present in large excess relative to the target metal species. We found that the equilibrium uptake of the material does not drop even after five adsorption–desorption cycles (Figure S18), suggesting that the material remains intact. As further support, we also carried out PDF analysis of Zr–Phytate after Pb^2+^ adsorption and desorption to assess how the local structure is affected by the regeneration treatment. The PDF of Pb-loaded Zr−Phytate (Figure 6a) shows small additional features at 2.1−2.7 Å, matching the P−O bond distances of monodentate and bidentate binding modes (Figure 6c). The features disappear after soaking the Pb-loaded Zr−Phytate in 1 M HCl solution, indicating complete desorption. We note that there is also an almost perfect match in the PDF of the regenerated material with the pristine as-synthesized Zr−Phytate, as seen in the difference curve in Figure 6a, indicating no change in the local structure due to the material’s high stability.

To test the boundaries of the materials’ stability, we soaked Zr–Phytate in 10 M HCl, HNO_3_, and H_2_SO_4_, and subsequently measured the carbon wt % using EA and Zr leaching using ICP-OES (Table S11). The results show that the chemical composition remains unchanged in the highly concentrated HCl and HNO_3_ solutions, demonstrating that these conditions can be reliably used for the materials regeneration. For instance, the carbon content went from 7.44(4) for the parent material to 7.59(6) and 7.49(1) wt % after soaking in HCl and HNO_3_, respectively. Furthermore, no leached Zr was detected in either acid (Table S11), confirming the material’s stability in these media. In H_2_SO_4_, on the other hand, a measurable amount of Zr in the leaching solutions was detected, indicating that Zr–Phytate is less stable in highly concentrated sulfuric acid. Sulfates, like phosphates, are oxyanions that form relatively strong bonds with Zr^4+^ cations and, thus, can displace phytic acid in the Zr–Phytate CP if they are present in sufficiently high concentrations. While the material is stable in highly acidic media, which is sufficient for the targeted application and subsequent regeneration, we note that under sufficiently basic conditions, Zr–Phytate starts to degrade. When placed in NaOH, the local structure of the sample at pH 10 remains almost identical to that of the pristine sample (Figure S19). At pH 12, the local structure starts to significantly differ from that of the pristine sample (Figure S19), and we observe Zr and ligand leaching (Table S11).

We believe these results show great potential for Zr–Phytate in real-world applications, as its building blocks are cheap and nontoxic, the material shows high Pb^2+^ uptake at low Pb^2+^ concentrations, even in highly competitive environments, and adsorbed Pb^2+^ can be reversibly desorbed using low-cost aqueous HCl solutions without adsorbent degradation.

## Conclusion

This work establishes the remarkable potential of an amorphous Zr–Phytate coordination polymer as an efficient adsorbent for water remediation applications. Through complementary PDF analysis and solid-state NMR, we elucidated the local structure of this amorphous material, revealing octahedral ZrO_6_ centers interconnected via phosphate groups—a motif reminiscent of that of τ-Zr­(HPO_4_)_2_. Zr-Phytate, synthesized under mild conditions using water at room temperature and inexpensive precursors, demonstrates exceptional Pb^2+^ adsorption performance at environmentally relevant concentrations. Most notably, it maintains extraordinary lead selectivity and capacity even in highly competitive environments where interfering ions exceed Pb^2+^ concentrations by more than 2 orders of magnitude. The material’s exceptional chemical durability in harsh acidic conditions renders it particularly attractive for practical applications. This stability enables facile regeneration through proton-exchange processes in acidic media, facilitating cost-effective recycling protocols. Critically, the adsorbent maintains consistent regenerative capacity across five consecutive cycles while preserving structural integrity, as confirmed by PDF analysis, with no detectable Zr leaching observed via ICP-OES measurements. When benchmarked against commercial ion-exchange resins, Zr–phytate exhibits markedly superior Pb^2+^ selectivity, underscoring its promise for real-world water treatment scenarios. The convergence of mild synthesis conditions, outstanding performance metrics, and robust regenerability positions this material as a compelling candidate for sustainable lead remediation technologies.

## Methods

### Synthesis

Ten mL portion of a 0.1 M ZrOCl_2_ solution was transferred into a 40 mL vial, followed by the addition of 10 mL of a *X* M Phytic acid solution (*X*; **1**: 0.1 M, **2**: 0.033 M, **3**: 0.017 M, and **4**: 0.011 M). The mixture was then shaken at room temperature for 3 h. Immediately upon the addition of PA, the formation of a white polymer was observed. The reaction time was extended to 3 h. Then, the white powder was isolated using a centrifuge and washed several times with distilled water until the pH of the supernatant reached around 6. The product was then dried in a vacuum oven for 1 day at 40 ^◦^ C. More details can be found in the Supporting Information.

### Adsorption Experiments

Batch adsorption experiments are generally conducted using an adsorbent dosage of 0.1 mg/mL. For isotherm studies, powder and solutions were added to a 20 mL glass vial and placed in a thermoshaker for 48 h. For kinetic experiments, 50 mg of powder was added to 500 mL of a lead solution, and 4 mL aliquots were taken at different time points. Solutions were filtered using PTFE membrane syringe filters with a mesh size of 0.22 μm, acidified with nitric acid, followed by analysis using ICP-OES. Solutions for selectivity experiments and adsorption experiments at low concentrations (below 1 ppm) were analyzed via ICP-MS and ICP-OES. Details can be found in the Supporting Information. The removal efficiency (*R*_e_), the adsorption capacity at a given equilibrium concentration (*c*_e_), and the distribution coefficient (*K*_d_) were calculated with the following equations:

1 $$R_{e}=\left(\frac{c_{0}-c_{e}}{c_{0}}\right)\times 100$$

2 $$q_{e}=\left(\frac{c_{0}-c_{e}}{m}\right)\times V$$

3 $$K_{d}=\frac{q_{e}}{c_{e}}$$

where *R*_e_ (%) is the removal efficiency, *c*_0_ and *c*_e_ are the Pb^2+^ concentration (mg/L) of the bulk solution before and after adsorption, respectively, *V* is the volume of the metal ion solution volume in liter, *m* is the amount of adsorbent (mg) added to the metal ion solution, and *q*_e_ (mg/g) is the amount of adsorbate (metal ions) in mg adsorbed per amount of adsorbent (g). *K*_d_ (L/g) gives the ratio of metal ions adsorbed to the amount of metal ions in the bulk solution at equilibrium. Details about how the fitting was done and the equations used for the adsorption models are provided in the Supporting Information.

### Characterization

*Nitrogen adsorption* measurements were performed on a Belsorp Max-II instrument at 77 K. *X-ray photoelectron spectroscopy (XPS)* measurements were carried out on a Kratos AXIS Supra instrument with an Al Kα X-ray source. Data fitting was done by using CasaXPS software. All data were calibrated to the Carbon 1s bond at 284.8 eV. *Thermogravimetric analysis (TGA)* profiles were obtained using a TA Q-Series TGA Q500. The balance flow rate was 15 mL/min with nitrogen. The thermal stability profile was measured in 15 mL/min airflow with a ramp of 1 °C per minute up to 1000 °C. *FTIR* spectra were collected using a PerkinElmer Frontier MIR/FIR spectrometer. Samples were pressed on a diamond window, and spectra were recorded between 4000 and 400 cm^–1^ at a resolution of 2 cm^–1^. *Powder X-ray diffraction* measurements were performed on a Bruker D8 Discovery instrument equipped with a Cu *K*_α_ source using a Bragg–Brentano geometry. In the incident beam, a divergent slit and a 2.5° axial Soller slit were installed. In the diffraction beam, a 2.5° axial Soller slit and a Ni absorber were installed to filter out the Cu *K*_β_ line. X-ray generator settings of 40 kV and 40 mA were employed. Powder patterns were collected with a step size of 0.02° 2θ and an acquisition time of 1 s per step. For PDF analysis, powder patterns were measured in a Debye–Scherrer geometry (transmission mode) using a Mo *K*_α_ X-ray tube (λ = 0.71073 Å) and a focusing Goebel mirror in the incident beam path. Data were collected using a variable count time protocol, which improves the counting statistics at high-*Q*. The protocols used are analogous to previous reports.^83^ Briefly, 5 scans were measured and subsequently accumulated into one single 1D diffraction pattern. This was then used for data reduction and calculation of *G*(*r*) using PDFgetX3.^84^ *Solid state* ^*31*^ *P nuclear magnetic resonance (ssNMR)* measurements were recorded on a 400 MHz Bruker spectrometer (9.4 T) equipped with an Avance III HD console and a 3.2 mm three-channel low-temperature magic-angle spinning probe. The sample was packed into a 3.2 mm zirconia rotor and spun up to 15 kHz spinning speed using dry nitrogen gas. The spectrum was recorded using cross-polarization (CP) with variable amplitude during a contact time of 2.0 ms.^85^ A linear ramp of 70–100% was applied at the ^1^H radio frequency field of 83 kHz while an 83 kHz pulse was applied on ^31^P and matched the first spinning sideband of the Hartmann–Hahn condition. 4096 transients were cumulated, and recycling delays were set to 2.0 s, which corresponds to 1.3 × *T*_1_ of the protons. The ^31^P chemical shifts were referenced relative to 85% H_3_PO_4_ using the secondary reference NH_4_H_2_PO_4_ at 1.33 ppm.^86^ The spectra were fitted using the DMFit software.^87^

### Total Scattering Measurements and PDF Analysis

Total scattering measurements were conducted at BM31/Swiss Norwegian Beamlines (SNBL) at the European Synchrotron Radiation Facility (ESRF) in Grenoble, France, under proposal A31-1-205 (https://doi.esrf.fr/10.15151/ESRF-ES-1213114713).^88^ The powdered samples were packed in glass capillaries with an inner diameter of 1 mm and a wall thickness of 0.01 mm. The samples were mounted on a capillary spinner downstream of the beam. A PILATUS3 X CdTe 2 M detector was placed at an angle of 15° to the beam direction downstream of the sample to maximize the accessible Q-range. For each sample, 20 images with an acquisition time of 1 min were collected. 2D diffraction images were averaged and integrated using PyFAI.^89^ An empty glass capillary was measured and subtracted from the 1D diffractograms before data reduction. Additionally, phytic acid sodium salt (CAS: 14306-25-3) was used as the reference material. Data reduction to *G­(r)* was done using PDFgetX3.^84^ The Fourier transform from *F*(*Q*) to *G*(*r*) was done using *Q*_max_ = 25 Å, *Q*_min_ = 1.0 Å, and *rpoly* = 1.2. The modeling of Zr–phytate with the structural model of τ-ZrP^34^ was done using PDFgui.^90^ PDFs of discrete Zr-phosphate structures extracted from the cif file were done within DiffPy-CMI.^91^ The cif file of τ-ZrP was downloaded from the crystallographic open database (COD) under the entry code 4322672.^34^

The extracted cluster models (xyz format) are available for download on Zenodo (see the *Data availability* statement). First, the PDFs of each cluster were calculated using *U*_iso_ = 0.004 Å^–2^ for all atoms. The calculated PDFs were then fitted to the dPDF as described in Figure 2 a as a linear combination (LC) and Python’s *curve_fit* function from *scipy.optimize*. The LC equation was defined as follows:

4 $$G_{obs}=\overset{i=14}{\underset{i=1}{\sum }}c_{i}G_{i}$$

where *c*_*i*_ are the component weights for each calculated PDF (*G*_*i*_) and *G*_obs_ is the experimental PDF. Each component’s weight was initialized with 1/14, and the fit was done over a range of *r*_min_ = 1.0 Å and *r*_max_ = 5.0 Å (Figure S11). The resulting two clusters with the highest component weights were then fitted to the dPDF using a two-phase fit model, where the parameters δ_2_ and separate *U*_iso_ parameters for the Zr, P, and O atoms were refined (Table S2).

### Density Functional Theory Calculations

We used the Density Functional Theory calculations with ORCA.^92,93^ All calculations were performed using the hybrid functional PBE0, with the D4 empirical dispersion correction and the C-PCM implicit solvation method with water as the solvent.^94–105^ The Karlsruhe x2c Basis Sets x2c-TZVPall were used to take into account the effect of spin–orbit coupling with the Exact 2-component (X2C) method.^106,107^ Geometries were optimized with an energy tolerance of 5 × 10^–6^ hartree and an RMS force tolerance of 1 × 10^–4^ hartree. For all systems, frequency analysis was performed after geometry optimization to ensure a local minimum. The reaction Gibbs free energy is then computed as the difference between the Gibbs free energies of products and reactants.^108–111^ We computed partial charges based on the real space partition of the electron density following the Minimal Basis Iterative Stockholder method.^76^

## Supplementary Material



## Data Availability

Experimental data presented in this work and DFT-optimized structures are openly available on Zenodo at: 10.5281/zenodo.15490007.

## Notes

Additional experimental details and materials, including a photograph of the experimental setup (fixed-bed adsorption experiment); table containing recent, well-performing MOFs, COFs, and POPs for lead removal (**Table S1**) with additional references;^112–118^ Supplementary results, including titration results, diffraction and spectroscopic data, and results from DFT calculations **Figures S1–S19** and **Tables S2–S11** (PDF)
