# Supplementary material for: Lead, Locked Away: Porous Zr–Phytate Coordination Polymers for Rapid and Selective Removal of Pb2+ from Water
Source: J Am Chem Soc. 2025 Dec 4;147(50):45936–50. doi: 10.1021/jacs.5c11825 (PMC12715787; doi:10.1021/jacs.5c11825)
Supplement: Supplementary file 1 [file ja5c11825_si_001.pdf]

Supplementary Information for:

Lead, Locked Away: Porous Zr-Phytate Coordination Polymer for Rapid and Selective Removal of Pb^{2+} from Water

Nazanin Taheri,[†,‡] Till Schertenleib,[†,‡] Timo M.O. Felder,[‡] Laura Piveteau,[¶] Beatriz Mouriño,[§] Berend Smit,[§] Mohammad Khaja Nazeeruddin,[¶] and Wendy L. Queen[*,‡]

†*The authors have contributed equally to this work*

‡*Laboratory for functional inorganic materials (LFIM), Institute of Chemical Sciences and Engineering (ISIC), École Polytechnique Fédérale de Lausanne (EPFL), 1951 Sion, Switzerland*

¶*Institute of Chemical Sciences and Engineering (ISIC), École Polytechnique Fédérale de Lausanne (EPFL), 1015 Lausanne, Switzerland*

§*Laboratory of molecular simulations (LSMO), Institute of Chemical Sciences and Engineering (ISIC), École Polytechnique Fédérale de Lausanne (EPFL), 1951 Sion, Switzerland*

E-mail: wendy.queen@epfl.ch

Contents

1 Materials **3**

2 Methods **3**

2.1 Synthesis of Zr-Phytate 3

2.2 Synthesis of $Zr(HPO_4)_2$ 4

2.3 Pb^{2+} batch adsorption experiments 4

2.3.1 Adsorbent Optimization Experiment 4

2.3.2 Isotherm study 5

2.3.3 Adsorption Kinetics Study 5

2.3.4 Adsorption kinetics study at low concentration 5

2.3.5 Selectivity experiments 6

2.3.6 Selectivity experiments at different ratios 6

2.4 Recyclability experiments of Zr-Phytate 7

2.5 Rapid flow through experiment 7

2.6 Fitting of Pb^{2+} adsorption data 8

2.7 Stability Test Procedure 9

2.8 Calculation of Phytic Acid to Zirconium Ratio in Zr-Phytate 10

2.9 Ion exchange capacity 11

3 Literature Comparison **13**

4 Supplementary Results **14**

4.1 Synthesis Screen 14

4.2 NaOH titration curve 15

4.3 N_2 Adsorption Isotherms 16

4.4 Basic Characterization of Zr-Phytate 18

4.5 PDF Fitting 20

4.6 Pb^{2+} Adsorption Data 22
4.7 Pb^{2+} Binding Mechanism 25
Exchange energies 27
Comparing monodentate cases 28
Comparing chelating cases 28
Comparing alternative cases 28
4.8 Flow through experiment 30
4.9 Regenerative Pb^{2+} capacity 31
4.10 Stability Tests 31

References **33**

1 Materials

$ZrOCl_2{\cdot}8H_2O$, phytic acid ((50% (w/w) in H2O), Pb^{2+}(II) acetate trihydrate, nickel(II) nitrate hexahydrate, copper(II) nitrate trihydrate, cadmium nitrate tetrahydrate, sodium chloride, and magnesium chloride hexahydrate were supplied from Sigma Aldrich company and used as received without further purification. The water used in this work was ultra-pure water (MilliQ, 18.2 MΩcm).

2 Methods

2.1 Synthesis of Zr-Phytate

A series of compounds with varying Zr/Phytic acid (Zr/PA) molar ratios was synthesized to find the most effective one for Pb^{2+} removal. We changed the Zr/PA molar ratio with the expectation that this would result in distinct structures, each possessing a different number of phosphate groups, which could potentially affect their performance in Pb^{2+}

uptake. For this purpose, four Zr-Phytate materials, named **1**, **2**, **3**, and **4**, were synthesized using the following protocol: 10 mL of a 0.1 M $ZrOCl_2$ solution was transferred into a 40 mL vial, followed by the addition of 10 mL of an X M PA solution (X; **1**: 0.1 M, **2**: 0.033 M, **3**: 0.017 M and **4**: 0.011 M). The mixture was then shaken at room temperature for 3 hours. After adding phytic acid, the immediate formation of a white precipitate was observed. The reaction time was extended to 3 hours to optimize the yield. After the reaction was completed, the white powder was separated using a centrifuge and washed multiple times with distilled water until the pH, monitored with pH paper, reached around 6. The product was then dried in a vacuum oven for one day at 40 °C. The drying and washing processes can affect the porosity of the obtained material with changes ranging from 30 m^2/g to 89 m^2/g. Zr-Phytate-1 formed in 34% yield based on the amount of Zr added to the reaction solution, compared to how much ended up in the product.

2.2 Synthesis of $Zr(HPO_4)_2$

10 ml of a 0.1 M $ZrOCl_2$ solution was transferred into a 40 ml vial, then 10 ml of a 0.1 M H_3PO_4 solution was added. The mixture was then shaken at room temperature for 3 hours. Upon the addition of PA, the rapid formation of a white powder was observed. The reaction time was extended to 3 hours to optimize the yield. After completion, the white powder was isolated using a centrifuge and washed several times with distilled water until the pH reached around 6. The product was then dried in a vacuum oven for one day at 40 °C.

2.3 Pb^{2+} batch adsorption experiments

2.3.1 Adsorbent Optimization Experiment

To find the optimal adsorbent for effective Pb^{2+} removal, a study was performed to assess the performance among Zr-Phytate-1, Zr-Phytate-2, Zr-Phytate-3, and Zr-Phytate-4. Each

adsorbent (5 mg) was added to a 20 mL solution containing Pb^{2+} at a concentration of 10 mg/L, followed by one hour of shaking at 250 rpm (rotations per minute) using a Thermo Scientific Max Q4450 orbital shaker at room temperature. After the adsorption process, solid particles were removed by filtration using a 25 mm hydrophilic PTFE membrane syringe filter with 0.22 0.22 μm pores. The Pb^{2+} concentration in the filtrate was analyzed using ICP-OES.

2.3.2 Isotherm study

The Pb^{2+} removal from aqueous solutions with various concentrations (5-100 mg/L) was performed by adding around 1 mg of adsorbent into 10 mL of Pb^{2+} solution, followed by 48 hours of shaking at 250 rpm at room temperature. To determine the Pb^{2+} concentration, solid particles were removed by filtration using a 25 mm hydrophilic PTFE membrane syringe filter with 0.22 μm pores, and the filtrate was then measured via ICP-OES. The obtained results were employed to determine the sorption isotherms.

2.3.3 Adsorption Kinetics Study

Adsorption kinetic experiments were performed over different adsorption times (1–2880 min) by adding 50 mg of adsorbent into a 500 mL solution containing Pb^{2+} at a concentration of 50 mg/L. The mixture was stirred at 250 rpm at room temperature. At specified time intervals, 4 mL samples were taken, and the solid particles were filtered out using a 25 mm hydrophilic PTFE membrane syringe filter with 0.22 μm pores. The filtrate was then measured via ICP-OES. The obtained results were employed to determine the sorption isotherms.

2.3.4 Adsorption kinetics study at low concentration

250 mg of the adsorbent was added into a 500 mL solution containing Pb^{2+} at a concentration of 1 mg/L. The adsorption times ranged from 10 to 200 seconds. The mixture was

stirred at 250 rpm at room temperature. At specified time intervals, 4 mL samples were taken, and the solid particles were filtered using a 25 mm hydrophilic PTFE membrane syringe filter with 0.22 μm pores. The filtered samples were then analyzed by ICP-MS and ICP-OES to quantify the Pb^{2+} concentration.

2.3.5 Selectivity experiments

The selectivity of Zr-Phytate and commercial adsorbents, Lewatit TP207 and DK-100, over other metals, such as nickel(II), copper(II), cadmium(II), magnesium (II), and sodium(I), was evaluated at (100 mg/L) concentrations in aqueous solutions. The experiments involved adding 10 mg of the adsorbent into a 10 mL solution containing a mixture of all mentioned metals with a contact time of 48 hours to reach equilibrium. The mixture was shaken at 250 rpm at room temperature. Subsequently, the solid particles were removed by filtration using a 25 mm hydrophilic PTFE membrane syringe filter with 0.22 μm pores. The filtered solution was then analyzed via ICP-OES to determine the concentration of each metal.

2.3.6 Selectivity experiments at different ratios

The experiments involved adding 10 mg of the adsorbent into a 40 mL solution containing a mixture of nickel(II), copper(II), cadmium(II), magnesium (II), Calcium (II), and sodium(I) with a contact time of 48 hours to reach equilibrium. The mixture was shaken at 250 rpm at room temperature. Subsequently, the solid particles were removed by filtration using a 25 mm hydrophilic PTFE membrane syringe filter with 0.22 μm pores. The filtered solution was then analyzed via ICP-OES to determine the concentration of each metal. In a solution with a 1:1 ratio, the concentration of lead was 1 mg/L, and the concentrations of the other mentioned metals were 1 mg/L. In a solution with a 1:10 ratio, the lead concentration remained 1 mg/L, while the other metal concentrations increased to 10 mg/L. In the solution with a 1:100 ratio, lead was still 1 mg/L, and the other metals were present at 100

mg/L.

2.4 Recyclability experiments of Zr-Phytate

To examine the regenerability of Zr-Phytate, the first cycle of Pb^{2+} adsorption-desorption was conducted as follows: **Adsorption**: Pb^{2+} was loaded by adding 200 mg of Zr-Phytate into a 40 mL solution containing Pb^{2+} (100 mg/L) with a contact time of 2 hours. Subsequently, the suspension was centrifuged, and the supernatant was filtrated using a 25 mm hydrophilic PTFE membrane syringe filter with 0.22 μm pores. The filtrate was then analyzed via ICP-OES to calculate the Pb^{2+} removal efficiency. **Desorption**: To regenerate the adsorbed Pb^{2+}, Zr-Phytate with Pb^{2+} loaded was subjected to two separate treatments in 40 ml of 1 M HCl for one hour each. It is important to note that after the first treatment with 1 M HCl, approximately 92% of the adsorbed Pb^{2+} was desorbed. After the second treatment, this percentage increased to 100%. After each treatment, the suspension was centrifuged, and the supernatant was filtrated using a 25 mm hydrophilic PTFE membrane syringe filter with 0.22 μm pores. The filtered solution was then analyzed via ICP-OES to calculate the desorption yield. After desorption, Zr-Phytate was dried using a vacuum oven overnight to prepare for the second adsorption-desorption cycle. The procedure of the second adsorption-desorption cycle was the same as the first one and was repeated for a total of five cycles.

2.5 Rapid flow through experiment

10 mg of adsorbent powder (Zr-Phytate) was added to a glass pipette (inner diameter approx. 1 cm) and packed below and on top with glass wool, to keep the powder bed fixed. Then, a plastic tube was connected to the broader end of the pipette, and the other end was passed through a peristaltic pump and added to the metal ion solution. A photograph with the packed glass pipettes and the set-up with the peristaltic pump is shown in Figure S17a-

b. 40 mL of the metal ion solution was pumped through the bed using a flow rate of 1 mL/min. The effluent was analyzed using ICP-OES. The removal percentage of each metal ion from the 40 mL solution was calculated and plotted in Figure 5. The experiment was done with two different metal ion solutions; one containing a 1:1 mixture of Pb:interfering-ion (1 mL/L each), and one containing a 1:100 mixture of Pb:interfering-ion (1 mg/L for Pb^{2+} and 100 mg/L for each interfering cation). We note that a total of 6 interfering cations were included in the solution, which means that the total excess of interfering cations (sum of all cations) was 1:6 and 1:600, respectively.

2.6 Fitting of Pb^{2+} adsorption data

The fitting of the Freundlich and Langmuir isotherm models and the kinetic models to the experimental data was done using nonlinear least squares regression model, specifically the *curve_fit* function implemented in Python's *scipy.optimize* libraries. The uncertainties given for the fitted parameters are calculated by taking the square-root of the sum of the diagonal matrix elements of the covariance matrix. The functions used for the fitting are listed below. The Freundlich equation is:

$$q_e = K_F \, c_e^{1/n} \tag{1}$$

where K_F and $\frac{1}{n}$ are the Freundlich coefficients. The Langmuir equation is:

$$q_e = \frac{K_L \, q_{max} \, c_e}{1 + K_L \, c_e} \tag{2}$$

where K_L is the Langmuir coefficient, q_{max} is the maximum theoretical uptake based on the Langmuir model. The units for the coefficients are given in Table 1. The kinetic data

were fitted using the Elovich model:

$$q_t = \frac{1}{b_E} ln(1 + a_E\, b_E\, t) \tag{3}$$

where q_t is the Pb^{2+} uptake at time=t in mg/g and a_E and b_E are the fitted Elovich parameters and t is time in minutes. The units for the Elovich parameters are listed in Table 1. For completion, the data was also fitted using pseudo-first order

$$q_t = q_e\,(1 - e^{-k_1 * t}) \tag{4}$$

and pseudo-second order kinetic models

$$q_t = \frac{q_e^2\, k_2\, t}{1 + q_e\, k_2\, t} \tag{5}$$

as well as the interparticle diffusion model

$$q_t = k_{diff}\,\sqrt{t} + C \tag{6}$$

where k_1, k_2, and k_{diff} are respective rate constants, q_e is the equilibrium uptake, in this case a fitted variable of the pseudo-second order model, and C is constant specific to the intraparticle diffusion model. The fitted results are shown in Table S3.

2.7 Stability Test Procedure

To evaluate the acid stability of Zr-Phytate, 10 mg of the material was soaked separately in 10 mL of 10 M solutions of HCl, HNO_3, H_2SO_4 for 24 hours. The stability in basic conditions was tested analogously in 0.0001, 0.01, and 0.1 M NaOH solutions. After soaking, the solutions were filtered using a 25 mm hydrophilic PTFE syringe filter with 0.22 μm pore size to remove any solid residues. To determine the concentration of zirconium

leached into the solution, 0.5 mL of the filtrate was diluted 20-fold with 2% HNO_3, and the resulting solution was analyzed by ICP-OES. The residual solid powder was washed once with deionized water and then dried under vacuum. The carbon content (wt.%) of the Zr-Phytate was measured both before and after acid soaking using elemental analysis. The initial %C of the pristine Zr-Phytate before soaking in acid was 7.44 ± 0.04%.

2.8 Calculation of Phytic Acid to Zirconium Ratio in Zr-Phytate

Elemental analysis was used to determine the carbon content (%C) of Zr-Phytate. To calculate the PA content, the molar mass of phytic acid (PA) (M_{PA}) was taken as approximately 660.04 g/mol. The following stoichiometry was applied:

$$\%\text{PA} = X * \left(\frac{M_{PA}}{F_{carbon}} \right) \tag{7}$$

where X is the mass of carbon (in grams) in 100 g of sample (i.e., %C), and F_{carbon} is the subtotal mass of carbon in the molecular weight formula of PA. This yields the estimated grams of PA per 100 g of sample.

To calculate the weight percentage of zirconium (Zr), Zr-Phytate was subjected to thermogravimetric analysis (TGA) by heating up to 1000 °C to convert the material to ZrP_2O_7. The percentage of Zr was calculated based on the residual weight at 1000 °C using the following formula:

$$\%\text{Zr} = m_r * \frac{M_{Zr}}{M_{product}} \tag{8}$$

where M_{Zr} is the atomic weight of Zr (91.224 g/mol) and $M_{product}$ is the molecular weight of the product formed at 1000 °C (ZrP_2O_7), which is 265.16 g/mol.

To obtain the PA:Zr mass-ratio, the calculated PA content (from elemental analysis) was divided by the Zr content obtained from TGA. The molar ratio is obtained by dividing each mass by the corresponding molecular weight (660.04 g/mol for PA, and 91.22 g/mol

for Zr). This gives us a molar PA:Zr ratio of ∼0.4.

2.9 Ion exchange capacity

We use a routine way to quantify the cation exchange capacity of ion exchange resins. It is a way to quantify the number of groups that are capable of entering an ion exchange reaction.[1] It yields the miliequivalent (meq.) of exchangeable ions per dry gram of material via titration. In the case of cation exchange resins, one has to first make sure that the powder is fully protonated. Then, the protonated powder is added to a solution containing a hard cation (often Na^+). Some of the protons on the powder will then be exchanged by the cations (Na^+) in solution. The released protons can be quantified via titration with NaOH. This will continuously consume the released protons, which prevents the establishment of a Na^+/H^+ equilibrium, and consequently, protons keep being released until the powder is fully saturated with Na^+ and all H^+ has been released. The point should be visible in the titration curve as a sharp increase in the pH of the solution. There are some obvious uncertainties. For example, there might be diffusion limitations, inaccessibility of certain exchange sites, unfavorable exchange equilibria, or hydrolysis of bonds in the material. The method is still useful to make comparisons across different ion exchangers, as it is a standard protocol used in the literature.[1]

Procedure: First, 300 mg of the Zr-Phytate powder was soaked in 50 ml of 0.1 M HCl. The suspension was then centrifuged, rinsed once with MQ water, and centrifuged again. Then, 50 mg of the wet powder (m_{wet}) was taken and dried in the vacuum oven overnight, and the dry mass was measured (m_{dry}). 250 mg of the wet powder was used for the titration. For this, it was dispersed in 50 mL of 0.1 M NaCl. The titration was performed using 0.01 M NaOH. The pH was measured continuously using a potentiostat. After each drop of NaOH was added, it was waited until the pH became constant. The titration curve is plotted in Figure S2. The inflection point (V_{infl}) was used to calculate the cation exchange

capacity as follows:

$$\frac{\text{meq. of cation exchange capacity}}{\text{gram of dry H-from}} = \frac{c_{NaOH} * V_{infl}}{0.25 * \frac{m_{dry}}{m_{wet}}} \tag{9}$$

Where c_{NaOH} is the concentration of the NaOH solution (10 mmol/L). The titration curve is shown below in Figure S2, and using $V_{infl} = 0.125$ L, $m_{wet} = 50.4$ mg, and $m_{dry} = 22.7$ mg, we calculate a cation exchange capacity of **11.1 meq./g**. The reported cation exchange capacities for crytalline and gelatinous $Zr(HPO_4)_2$ samples are in the range of 5.81-6.71 meq./g.[2]

3 Literature Comparison

Table S1: Literature overview: Materials discussed in the introduction and more recent (past 5 years) adsorbents for Pb^{2+} removal from water. The listed materials were included in this table based on their high reported Pb^{2+} capacities. Papers that include reusability experiments were prioritized. The listed maximum Pb^{2+} uptake (q_{max}) was either obtained from an adsorption experiment, or it is the fitted parameter from the Langmuir model. If the latter is the case, the associated K_L constant is also reported.

Class	Material	Year	q_{max} (mgg^{-1})	K_L (L mg^{-1})	$q_{max}K_L$ (L/g)	Reusability	Notes	Stability
CP	Zr-Phytate	2025	427	69	29 000	5 cycles	This work	—
Resin	TP207	2025	612	5.4	3 300	—	This work	—
Resin	TS260	2025	418	18	7 000	—	This work	—
Resin	DK100	2025	541	0.3	190	—	This work	—
MOF	Zn-4,4′-azoxydibenzoate	2017 [3]	616.64			5 cycles with drop of performance	High density of specific adsorption sites (O^- groups)	Changes in structure and loss of crystallinity are obvious in the data, but not discussed in the paper.
MOF	MIL-53@NP	2015 [4]	492.4			No reusability experiments done.	MOF combined with magnetic nanoparticles.	Al leaching during cycling.
MOF	Cu-BDC	2019 [5]	333.3	0.3896	130	3 cycles, with incomplete (70%) desorption using water & drop of capacity of ∼30%.		Proposed adsorption sites are COO^- groups of the BDC linkers. This implies that Cu^{2+} from the metal node should be displaced by Pb^{2+}. Inconclusive stability tests.
MOF	Zn-BDC	2019 [5]	312.5	1.882	588	3 cycles, with incomplete (70%) desorption using water & drop of capacity of ∼50%.		Proposed adsorption sites are COO^- groups of the BDC linkers. This implies that Zn^{2+} from the metal node should be displaced by Pb^{2+}. Inconclusive stability tests.
MOF	HCMUE-2	2024 [6]	1309	0.004	5	7 cycles; regenerative capacity not reported	OH-rich Zr–MOF	Loss of crystallinity after regeneration is evident in the PXRD pattern. Lead desorption was not confirmed during cycling.
MOF	UiO-66-TCA	2025 [7]	968	0.0083	8	>20% of ads. capacity after 5 cycles	Tricarboxylic acid functionalized UiO-66 derivative	Gradual loss of crystallinity and reduced adsorption capacity after 5 cycles.
MOF	UiO-66-PTC	2021 [8]	209	0.059	12	20% drop after 4 cycles	Phenylthiosemicarbazide–functionalized UiO-66-NH_2; High selectivity with K_d = 18 L/g.	Desorption with thiourea. Chemical degradation during regeneration.
POP	NR-POP	2023 [9]	833	0.1016	85	4 cycles, desorption not reported.	N-rich triazine POP	No informaiton available.
POP	TPABPOP-1	2023 [10]	472	0.14	66	—	Amine Functionalized POP; BET 1290 m^2g^{-1}	No informaiton available.
COF	TAVA-S-Et-SH	2024 [11]	330	0.0061	2	5 cycles, desorption not reported.	Selective against Cu, Ni, Zn, Cd. Thiol groups as ads. sites.	No informaiton available.
COF	COF-OH/CS	2025 [12]	1268	0.0061	8	—	COF-OH mixed with chitosan	No informaiton available.
COF	BTT-DHBD-COF	2025 [13]	572	0.116	66	15% loss after 5 cycles, incomplete desorption.	Ortho-N–S / N–O dual-site design	No information available.

4 Supplementary Results

4.1 Synthesis Screen

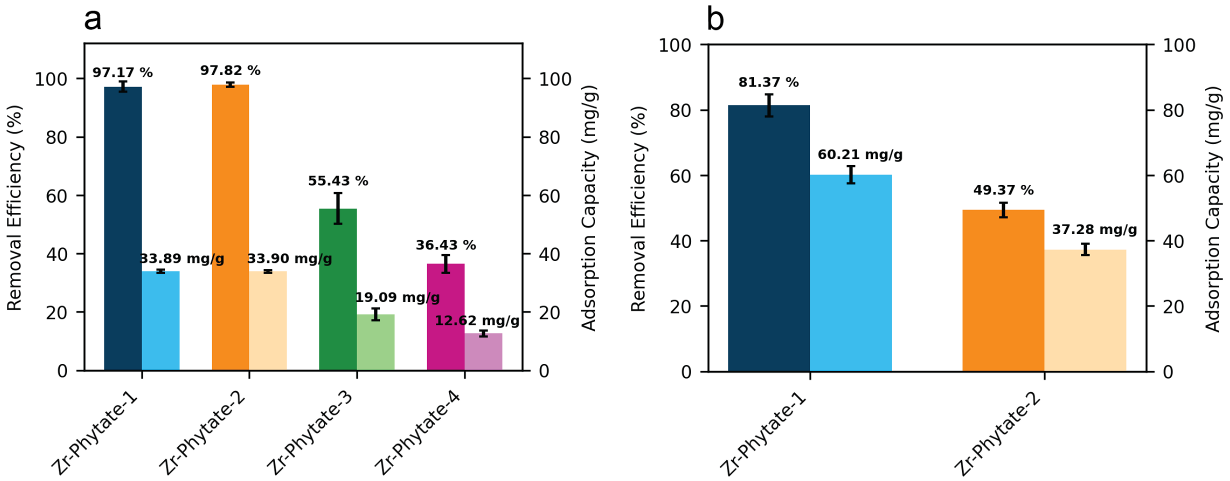

Figure S1: Bar plots showing Pb^{2+} removal efficiency (%) and adsorption capacity (mg/g) of Zr-Phytate (1-4) from aqueous solution at room temperature. (condition: (a): Adsorbent dosage: 5 mg, the Pb^{2+} solution volume: 20 mL, and adsorption time: 1 hour, (b): Adsorbent dosage: 5 mg, the Pb^{2+} solution volume: 40 mL, and adsorption time: 1 hour, adsorbent: Zr-Phytate (1) and (2).

4.2 NaOH titration curve

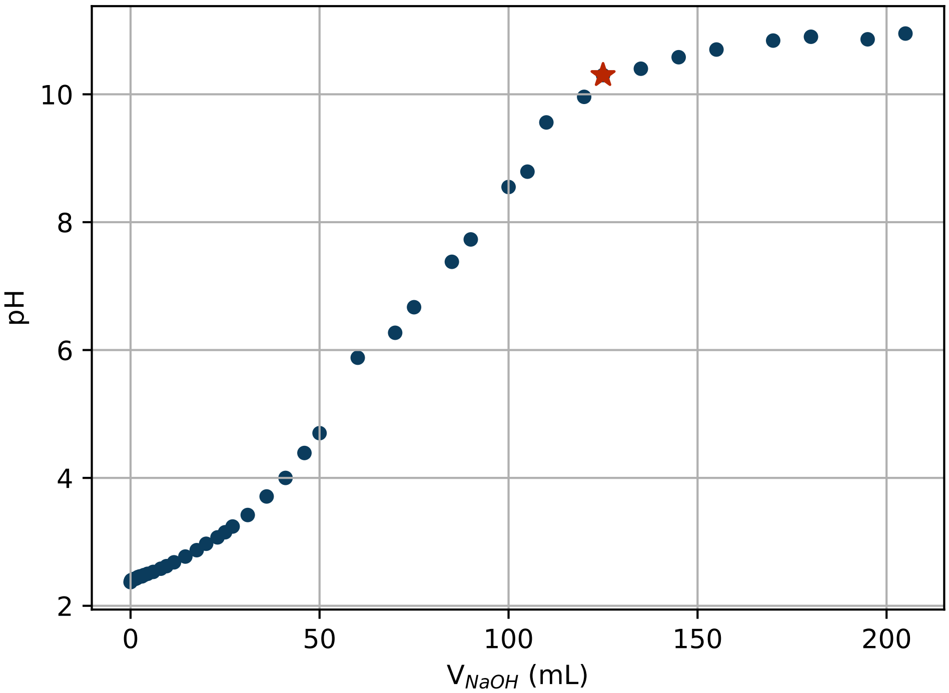

Figure S2: Titration curve to determine the cation exchange capacity of Zr-Phytate. A 10 mM NaOH solution was used for the titration, and the pH was measured using a pH meter. The endpoint is marked with a start at $V = 125$ mL. The curve is used to determine the ion exchange capacity of Zr-Phytate as described in Section 2.9.

4.3 N_2 Adsorption Isotherms

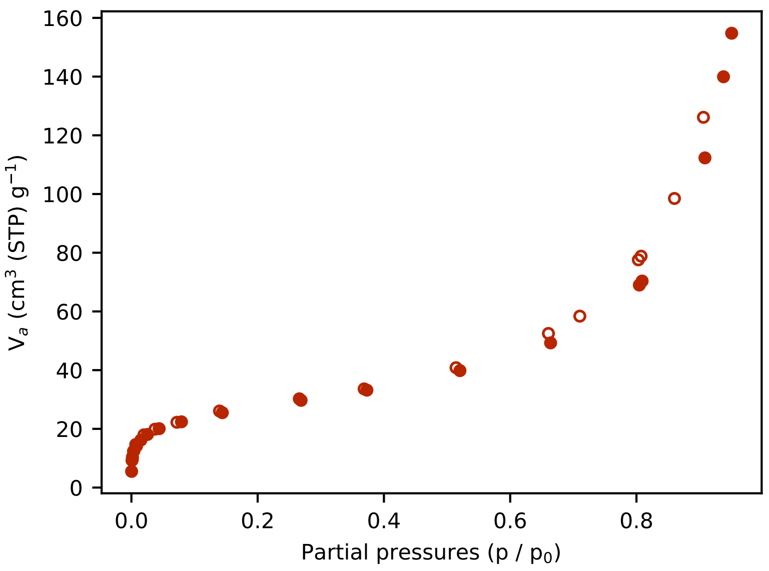

Figure S3: N_2 adsorption isotherm of Zr-Phytate (second batch). The first batch is plotted in the main manuscript.

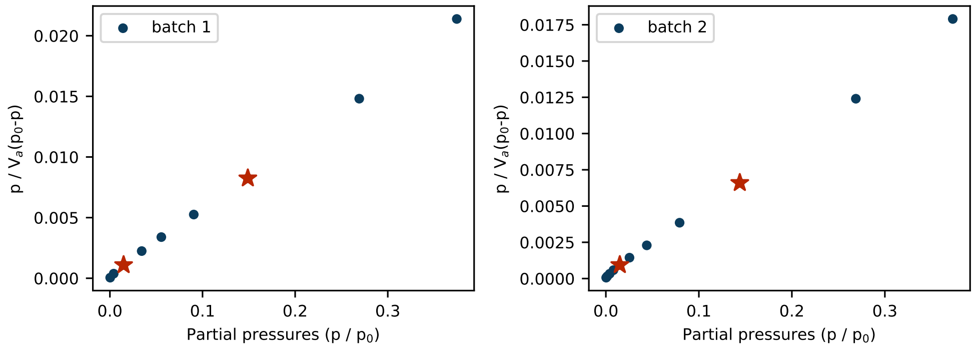

Figure S4: BET plot showing the start and end point for the BET calculation (highlighted with red stars) for two independent batches of Zr-Phytate.

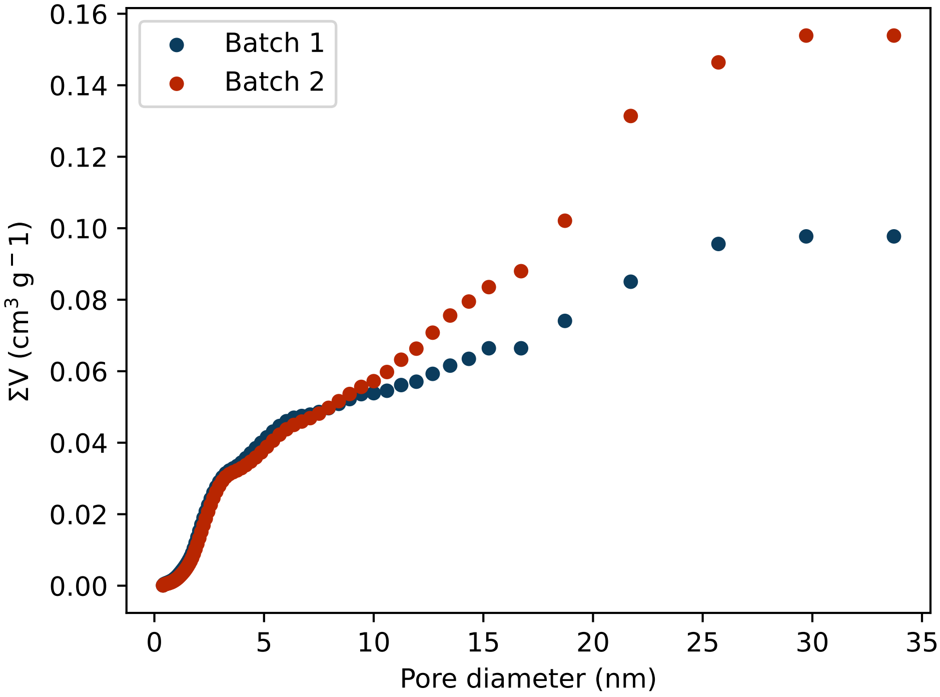

Figure S5: Non-local density functional theory (NLDFT) cumulative pore size distribution calculated from N_2 isotherms at 77 K of two separately synthesized batches of Zr-Phytate. The pore size distribution may vary slightly between different synthesis batches; the materials' porosity appears to be highly sensitive to the drying procedure, i.e., the amount of water in the wet powder prior to drying.

4.4 Basic Characterization of Zr-Phytate

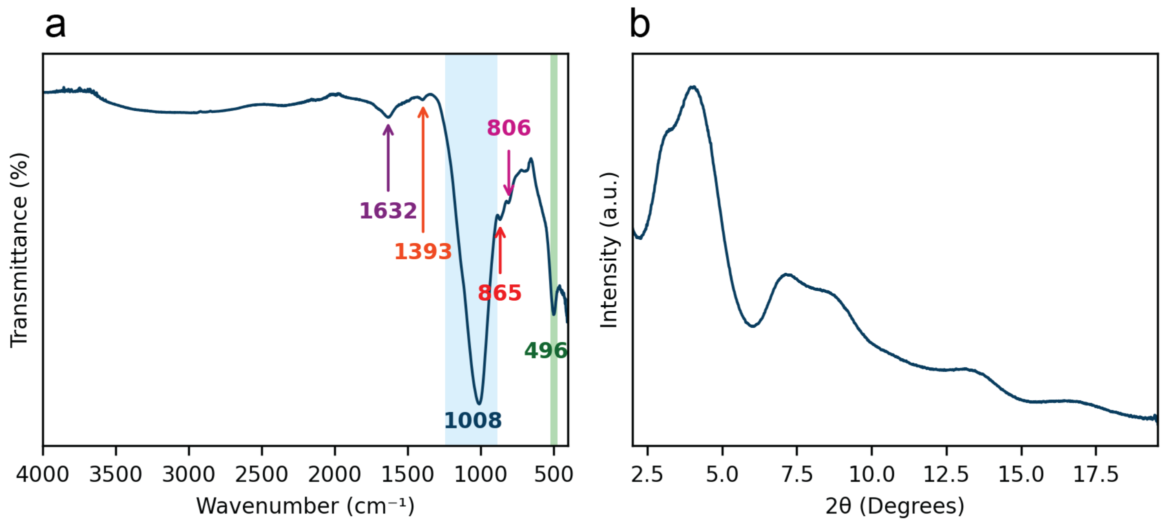

Figure S6: (a) FTIR spectra, (b) PXRD pattern of Zr-Phytate (λ = 0.24484 Å).

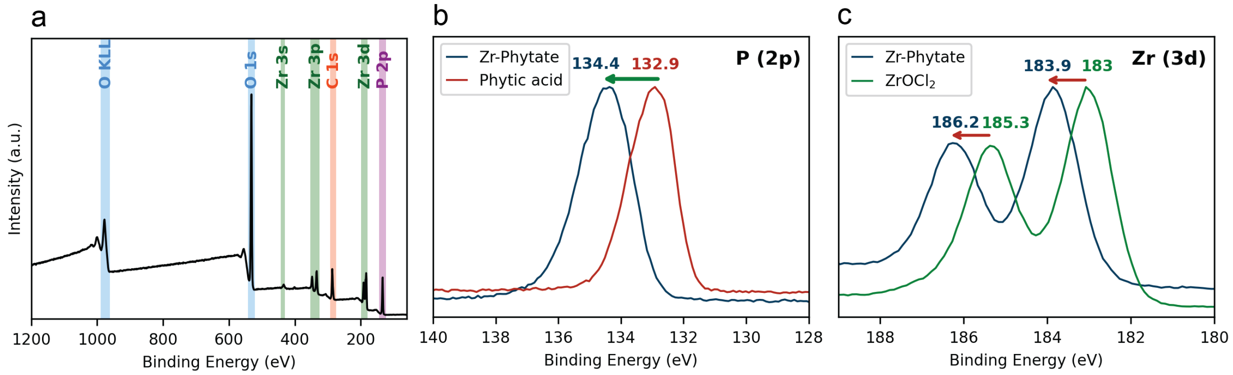

Figure S7: (a) Wide XPS spectrum, (b) High-resolution XPS spectrum of Zr 3d, and (c) High-resolution XPS spectrum of P 2p of Zr-Phytate.

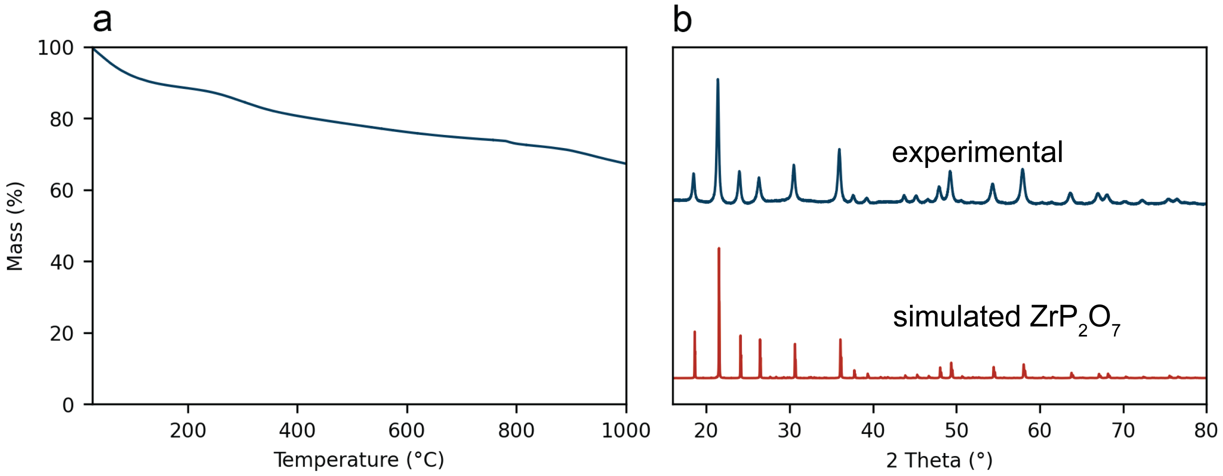

Figure S8: (a) Thermogram and (b) PXRD pattern of Zr-Phytate heated up to 1000 °C. Below in red, the simulated PXRD pattern of ZrP_2O_7 is plotted, and on top in blue, the experimental PXRD of Zr-Phytate after heating to 1000 °C.

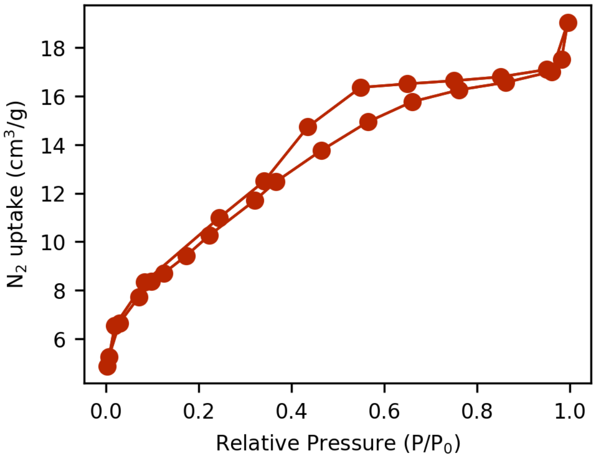

Figure S9: N_2 adsorption isotherm of $Zr(HPO_4)_2$ measured at 77 K.

4.5 PDF Fitting

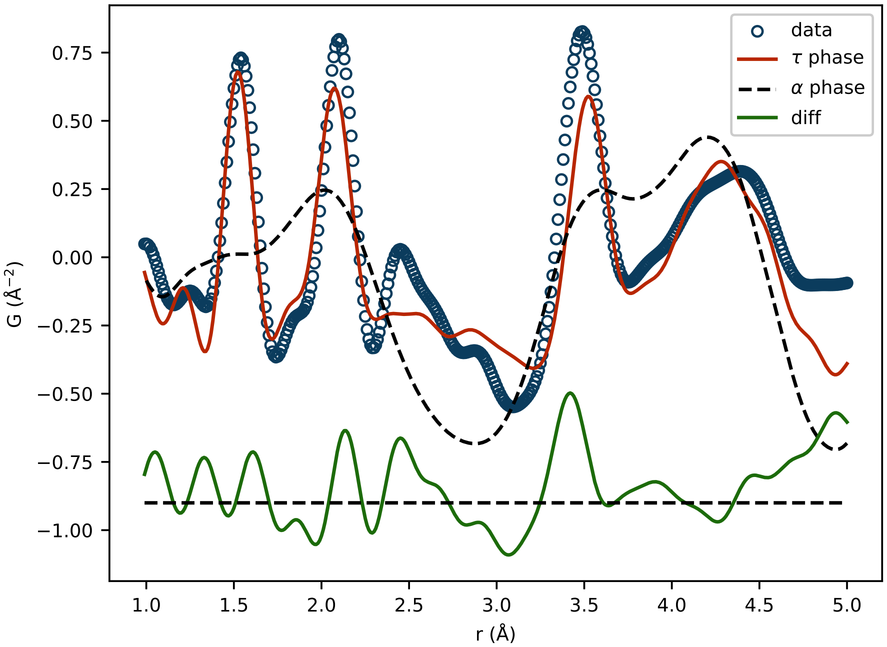

Figure S10: Fitting result of Zr-Phytate using the structural model of τ-ZrP (red). Although the model fits the PDF poorly (difference curve in green), the three main features, which correspond to P-O, Zr-O, and Zr-P peaks, are reproduced relatively well. As a comparison, the PDF of the α phase of ZrP is plotted as well (black, dashed lines), showing that it does not have a similar local structure like Zr-Phytate.

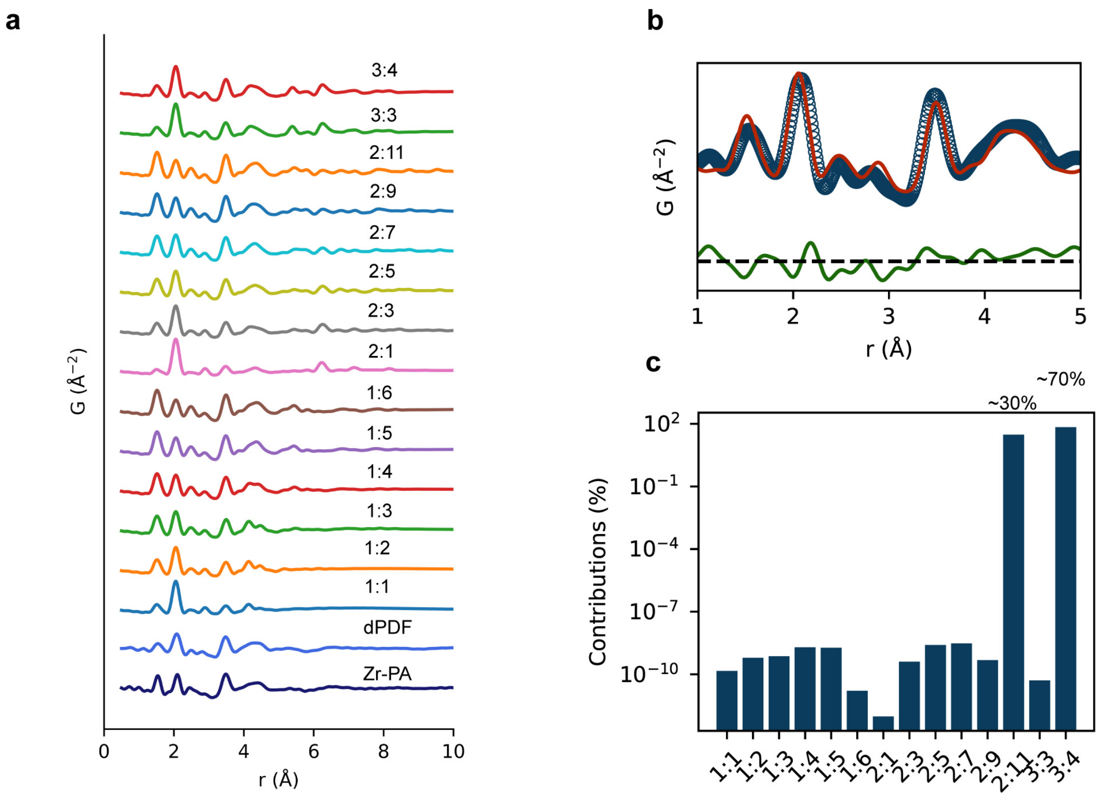

Figure S11: (a) A set of discrete Zr-phosphate structural models was extracted from the CIF file of τ-ZrP. The models were chosen to have a set of models with varying ratios of Zr and phosphate. The relative amount of those influences the relative intensities of the peaks around 1.5 Å (P-O), 2 Å (Zr-O), and 3 Å (Zr-P). The scattering contribution from phytic acid was removed by subtracting the PDF of sodium phytate from the PDF of Zr-Phytate. (b) The resulting differential PDF (dPDF) was fitted using a linear combination of the discrete Zr-phosphate structures, and (c) shows the relative contributions of each model to the linear combination.

Table S2: Refined parameters for two-hase model fit (Figure 2 d) where phase 1 was $Zr_3(PO_4)_4$ and phase two was $Zr_2(PO_4)_{11}$. The zoomscale parameter was not refined independently, as it was set equal for both phases.

$R_w = 0.33$	$Zr_3(PO_4)_4$	$Zr_2(PO_4)_{11}$
scale	0.598	0.402
δ_2	1(6)	1(10)
Zr U_{iso}	0.001(25)	0.000010(10)
O U_{iso}	0.010(35)	0.03(14)
P U_{iso}	0.009(95)	0.0(1)
zoomscale	1.002(8)	1.002(8)

4.6 Pb^{2+} Adsorption Data

Table S3: Fitted parameters of different models from the kinetic plot

Model	Pseudo-first-order			Pseudo-second-order			Intraparticle diffusion		
	K_1	q_e	R^2	K_2	q_e	R^2	K_{diff}	C	R^2
Zr-Phytate	0.55	344.61	0.92	0.002	375.01	0.96	59.50	78.19	0.88

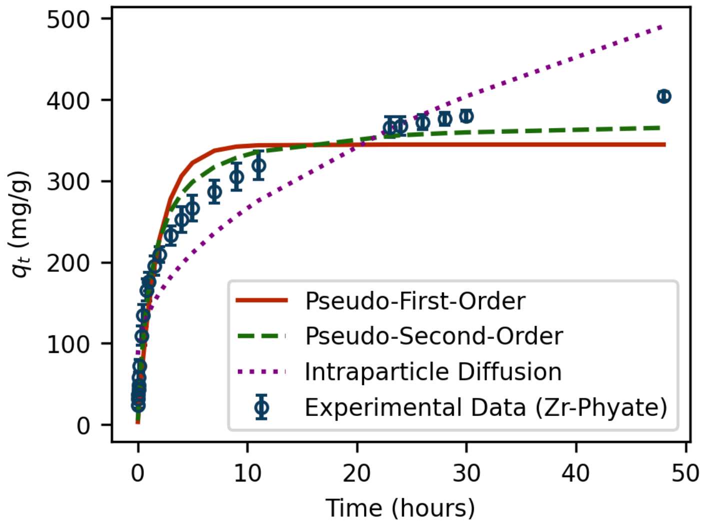

Figure S13: Kinetic experiment of Zr-Phytate (Pb^{2+} concentration: 50 mg/L, adsorbent dosage: 50 mg, volume: 500 mL, time: 48 h).

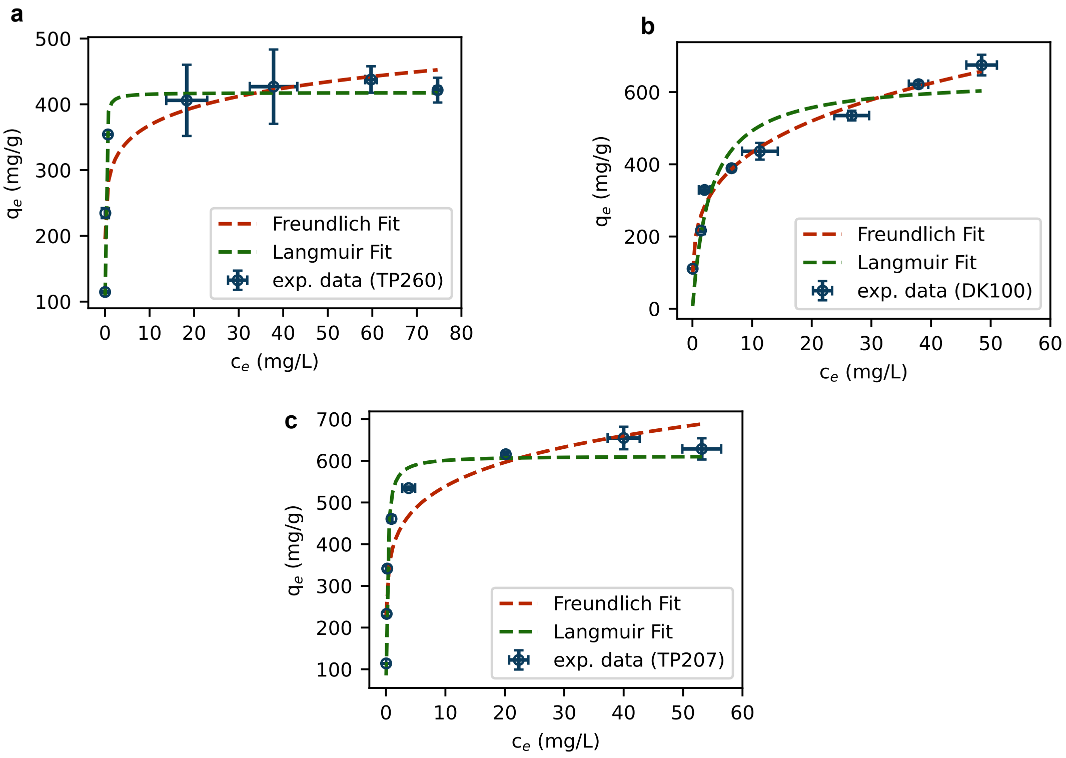

Figure S12: Pb^{2+} adsorption isotherms measured for commercial ion-exchange resins, including TP-260 (a), DK-100 (b), and TP-207 (c). To each set of experimental data, the Langmuir and the Freundlich adsorption isotherms were fitted. The fitted parameters are listed in Table 1 in the main manuscript.

Table S4: Measured cation concentrations via ICP-OES for the selectivity experiments plotted in Figure 3d.

	Wavelength (nm)	c_0 (mg/L)	c_e (mg/L)	stand. dev.
Pb	220.353	106.28	4.61	1.59
Cu	224.7	92.74	77.71	0.67
Cd	326.105	103.79	95.44	0.93
Ni	231.096	94.96	94.55	0.54
Na	589.592	128.87	126.80	0.48
Mg	280.27	102.79	77.71	0.67

Table S5: Measured cation concentrations via ICP-OES for the selectivity experiments plotted in Figure 4a.

	Wavelength (nm)	c_0 (mg/L)	c_e (mg/L)	stand. dev.
Pb	220.353	1.21	0.01	0.01
Cu	224.7	1.27	0.01	0.01
Cd	326.105	1.04	0.03	0.01
Ni	231.096	1.20	0.47	0.04
Ca	422.673	1.02	0.14	0.02
Na	589.592	0.51	0.66	0.01
Mg	280.27	1.71	0.64	0.06

Table S6: Measured cation concentrations via ICP-OES for the selectivity experiments plotted in Figure 4b.

	Wavelength (nm)	c_0 (mg/L)	c_e (mg/L)	stand. dev.
Pb	220.353	1.07	0.01	0.01
Cu	224.7	11.85	4.60	0.30
Cd	326.105	10.24	5.20	0.26
Ni	231.096	11.17	11.06	0.03
Ca	422.673	10.33	7.57	0.16
Na	589.592	5.86	6.08	0.03
Mg	280.27	14.81	14.43	0.06

Table S7: Measured cation concentrations via ICP-OES for the selectivity experiments plotted in Figure 4c.

	Wavelength (nm)	c_0 (mg/L)	c_e (mg/L)	stand. dev.
Pb	220.353	1.02	0.07	0.01
Cu	224.7	105.61	95.60	2.03
Cd	326.105	98.41	94.73	1.07
Ni	231.096	101.92	104.58	0.50
Ca	422.673	82.13	82.90	0.52
Na	589.592	67.83	69.40	0.48
Mg	280.27	73.92	75.94	0.49

4.7 Pb^{2+} Binding Mechanism

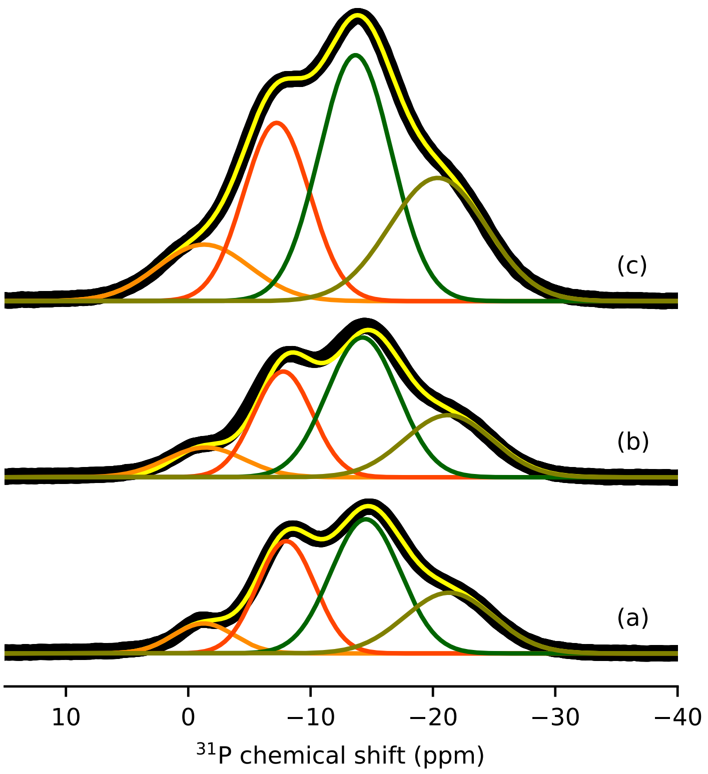

Figure S14: ^{31}P NMR of (a) as-synthesized Zr-Phytate, (b) loaded with 8 wt.% of Pb, and (c) with 15 wt.% of Pb. Legend: black (data), yellow (fit).

Table S8: The positions, widths, and contribution of the fitted Gaussians used to fit the ^{31}P NMR in Figure S14.

	Amplitude (a.u.)	Position	Width (ppm)	Line Shape	Integral	Assignment
as-synth.	433	-1.3 ppm	5.9	Gaussian	8%	Q0
	1621	-8.0 ppm	5.6	Gaussian	28%	Q1
	1938	-14.5 ppm	6.7	Gaussian	41%	Q1
	878	-21.3 ppm	8.5	Gaussian	23%	Q2
8 wt.% Pb	430	-1.4 ppm	7.5	Gaussian	9%	Q0
	1528	-7.8 ppm	5.6	Gaussian	26%	Q1
	2019	-14.2 ppm	6.9	Gaussian	42%	Q1
	898	-21.3 ppm	8.4	Gaussian	23%	Q2
15 wt.% Pb	817	-1.3 ppm	8.9	Gaussian	11%	Q0
	2575	-7.2 ppm	6.3	Gaussian	25%	Q1
	3552	-13.7ppm	6.9	Gaussian	38%	Q1
	1780	-20.4 ppm	9.3	Gaussian	26%	Q2

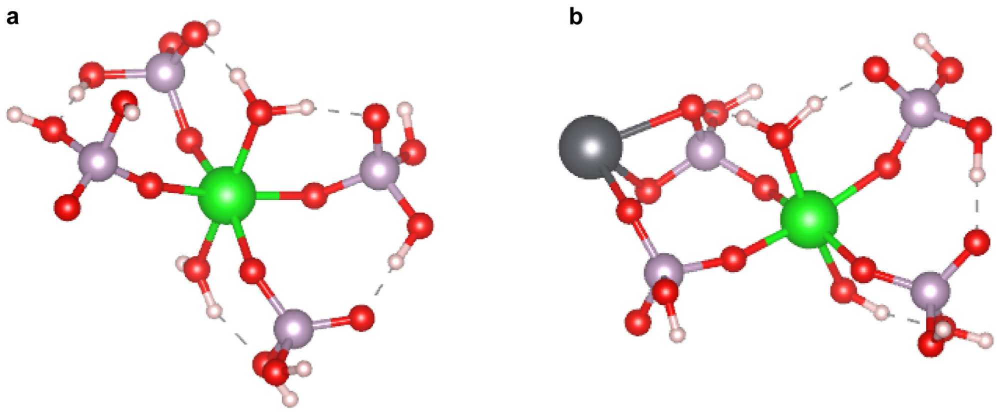

Figure S15: Structural models used to calculate charges in a zirconium-phosphate cluster before and after Pb^{2+} adsorption. (a) model cluster before Pb^{2+} adsorption: $Zr(H_2O)_2(H_2PO_4)_4$. (b) model cluster after Pb^{2+} adsorption: $Zr(H_2O)_2(H_2PO_4)_2Pb(HPO_4)_2$. The computed charges are tabulated in Table S9. Color code: green (Zr), red (O), dark gray (Pb), light gray (P), white (H).

Table S9: MBIS analysis for the two model structures in Figure S15.

System	Atom	Description	Charge	Population
$Zr(H_2O)_2(H_2PO_4)_4$	H	-	0.56	0.44
	H	-	0.56	0.44
	Zr	-	2.65	37.35
	P	Monodentate binding	2.04	12.96
	P	Chelation	2.04	12.96
	O	Chelation	-0.89	8.89
	O	Monodentate binding	-0.92	8.92
	O	Chelation	-1.02	9.02
$Zr(H_2O)_2(H_2PO_4)_2Pb(HPO_4)_2$	Pb	-	1.31	80.69
	Zr	-	2.64	37.36
	P	Monodentate binding	2.01	12.99
	P	Chelation	1.97	13.03
	O	Chelation	-0.93	8.93
	O	Monodentate binding	-0.95	8.95
	O	Chelation	-1.00	9.00

Exchange energies

Table S10: Computed Gibbs free energy for the systems in Figure S16.

System	Energy (Hartree)
AcO^-	-457.8868440200
$H_2PO_4{}^-$	-873.7955134200
$HPO_4{}^{2-}$	-873.3289671500
AcOH	-458.3324354300
$[Pb–AcO]^+$ (mono)	-21576.9813387900
$[Pb–H_2PO_4]^+$ (mono)	-21992.8931130500
Pb-HPO_4 (mono)	-21992.4441087500
$[Pb–AcO]^+$ (chelating)	-21576.9863753400
$[Pb–H_2PO_4]^+$ (chelating)	-21992.8861245200
Pb-HPO_4 (chelating)	-21992.4564238800

Comparing monodentate cases

R_1: $[Pb–AcO]^+ + H_2PO_4^- \rightarrow [Pb–H_2PO_4]^+ + AcO^-$ ΔG° = -9.152 kJ/mol

Going from Pb-acetate monodentate to $[Pb–H_2PO_4]^+$ monodentate.

R_2: $[Pb–AcO]^+ + H_2PO_4^- \rightarrow Pb\text{-}HPO_4 + AcOH$ ΔG° = 0.809 kJ/mol

Going from Pb-acetate monodentate to Pb-HPO_4 monodentate.

Comparing chelating cases

R_3: $[Pb–AcO]^+ + H_2PO_4^- \rightarrow [Pb–H_2PO_4]^+ + AcO^-$ ΔG° = 23.420 kJ/mol

Going from Pb-acetate chelating to $[Pb–H_2PO_4]^+$ chelating.

R_4: $[Pb–AcO]^+ + H_2PO_4^- \rightarrow Pb\text{-}HPO_4 + AcOH$ ΔG° = -18.301 kJ/mol

Going from Pb-acetate chelating to Pb-HPO_4 chelating.

Comparing alternative cases

R_5: $[Pb–AcO]^+ + H_2PO_4^- \rightarrow [Pb–H_2PO_4]^+ + AcO^-$ ΔG° = 5.072 kJ/mol

Going from Pb-acetate chelating to $[Pb–H_2PO_4]^+$ monodentate.

R_6: $[Pb–AcO]^+ + H_2PO_4^- \rightarrow Pb–HPO_4 + AcOH$ ΔG° = -18.301 kJ/mol

Going from Pb-acetate chelating to Pb-HPO_4 chelating.

R_7: $[Pb–H_2PO_4]^+ + HPO_4^{2-} \rightarrow Pb–HPO_4 + H_2PO_4^-$ ΔG° = -78.390 kJ/mol

Going from Pb-H_2PO_4 monodentate to Pb-HPO_4 chelating.

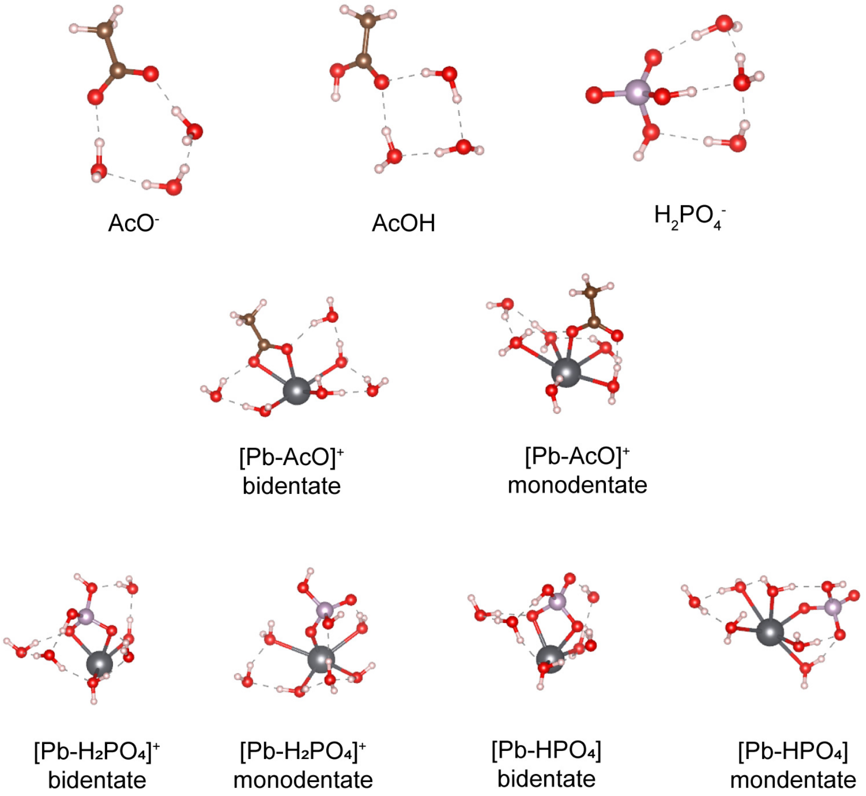

Figure S16: DFT-optimized structures used to compute the Gibbs free energies of the exchange reactions above. Color code: red (O), brown (C), light gray (P), white (H), dark gray (Pb).

4.8 Flow through experiment

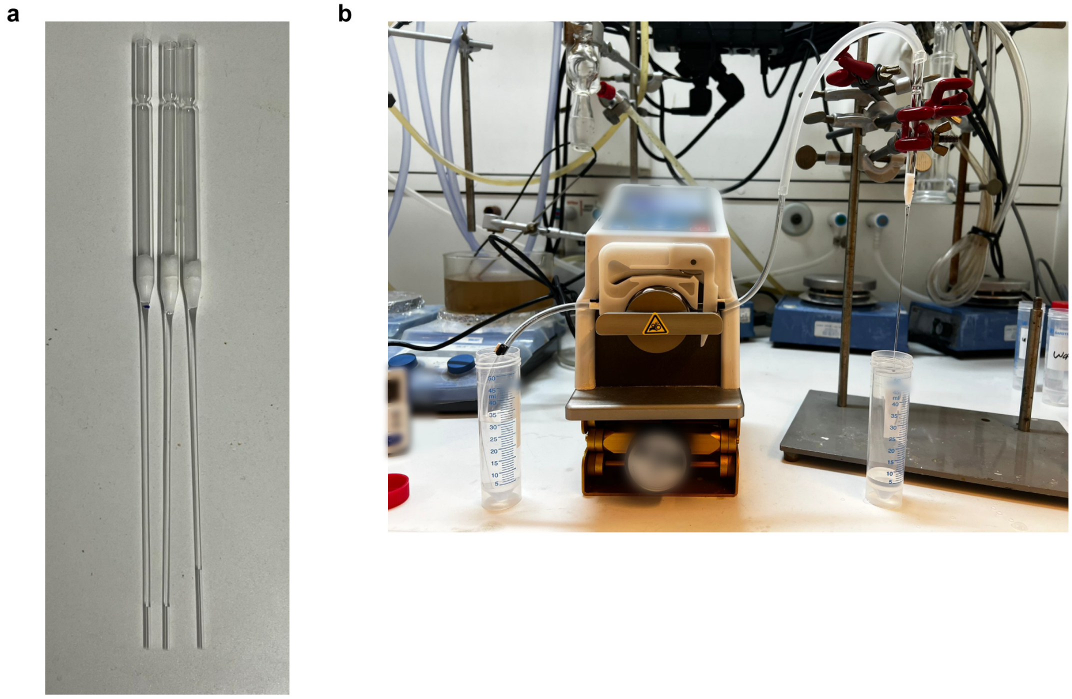

Figure S17: (a) photograph of glass pipettes filled with the adsorbent powder (Zr-Phytate) and packed below and on top with glass wool to fix the adsorption bed in place. (b) The setup used for the flow-through adsorption experiment. The photograph shows the peristaltic pump used to flow the metal ion solution from a 50 mL centrifuge tube through plastic tubing and through the glass pipette that contains the fixed adsorbent bed. The outlet after adsorption was collected in another 50 mL centrifuge tube. Parts of the image were blurred out to hide any company logos.

4.9 Regenerative Pb^{2+} capacity

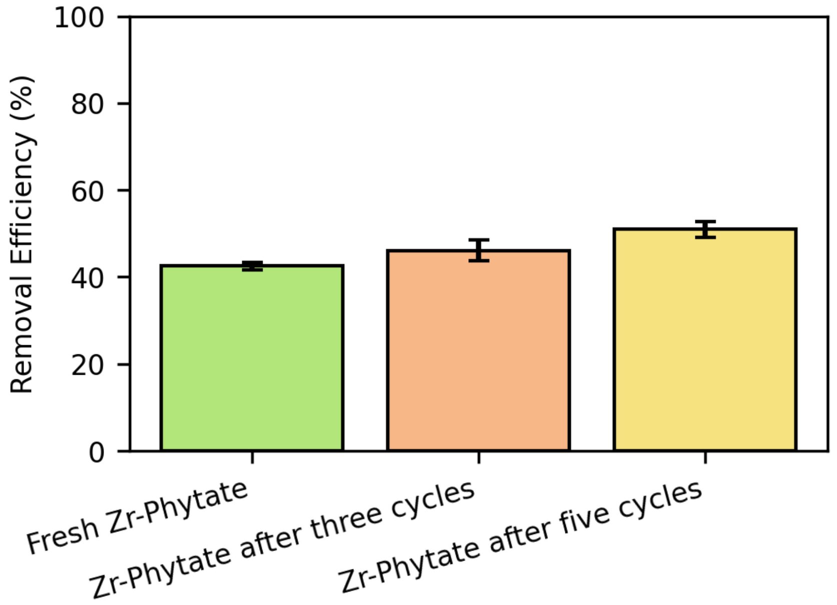

Figure S18: The regenerative capacity of Zr-Phytate was tested after three and five adsorption-desorption cycles using an adsorbent dosage of 0.1 mg/mL and an initial Pb^{2+} concentration of c_0 = 100 mg/L.

4.10 Stability Tests

Table S11: ICP-OES data of soaked Zr-Phytate under acidic and basic conditions and percentage carbon (%C) content of the soaked sample obtained from elemental analysis.

	Zr (mg/L)	wt% Zr leached	%C	% PA
10 M HCl	0.02	0.02	7.59 ± 0.06	69.5 ± 0.6
10 M HCl (Blank)	0.6			
10 M HNO_3	<LOD	<LOD	7.5 ± 0.1	68.6 ± 0.9
10 M HNO_3 (Blank)	<LOD			
10 M H_2SO_4	8.4 ± 1.8	0.84 ± 0.18	7.5 ± 0.6	41 ± 5
10 M H_2SO_4 (Blank)	2			
0.01 M NaOH	9.5 ± 0.9	0.95 ± 0.09	6.4 ± 0.1	58 ± 1
0.01 M NaOH (Blank)	0.0035			
0.1 M NaOH	0.010 ± 0.002	0.0010 ± 0.0002	3.5 ± 0.9	32 ± 8
0.1 M NaOH (Blank)	0.0014			

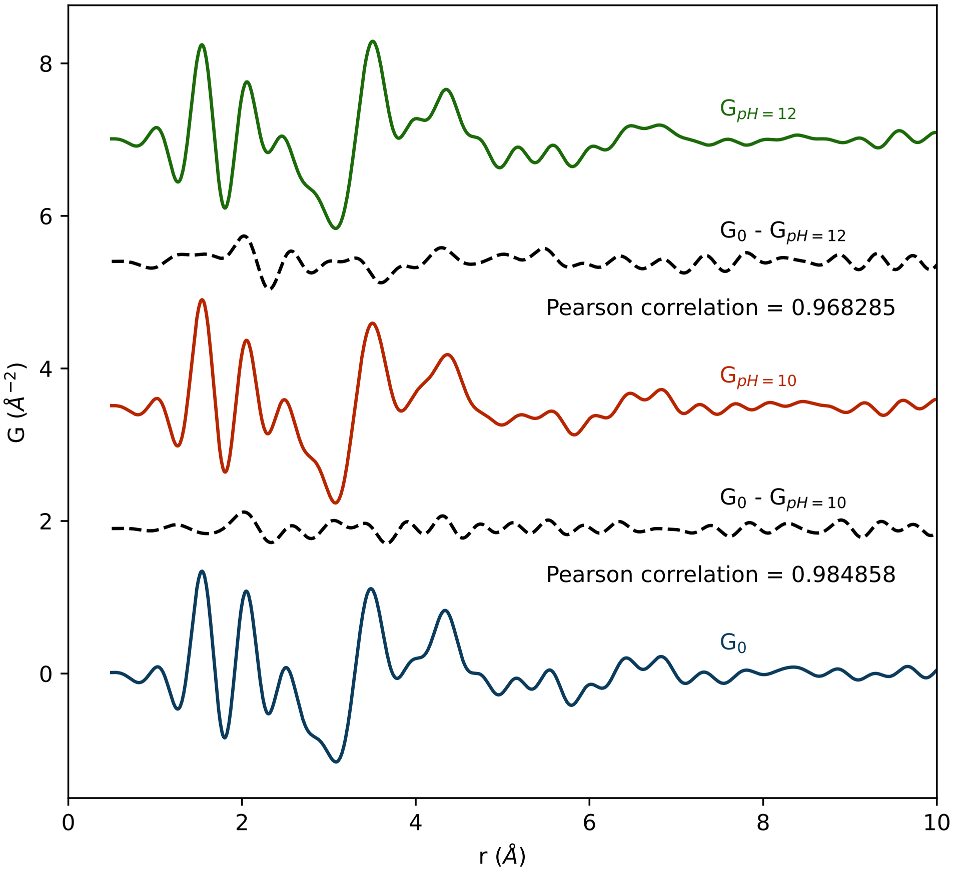

Figure S19: PDFs obtained from PXRD data measured using a Mo K_α source. Details about the measurement are provided in the *Method* section of the main article. PDFmorph was used to scale the PDFs of the NaOH-soaked samples ($G_{pH=10}$ and $G_{pH=12}$) to the PDF of the as-synthesized powder (G_0), to minimize the difference between the two. The rationale for this can be found in Schertenleib *et al.*[14] Solid lines are the PDFs, and the dashed lines are the differences between the NaOH-soaked samples ($G_{pH=10}$ and $G_{pH=12}$) and the as-synthesized powder (G_0). The Pearson correlation is given for both cases in the Figure below the corresponding difference curves

References

(1) Fisher, S.; Kunin, R. Routine Exchange Capacity Determinations of Ion Exchange Resins. *Analytical Chemistry* **1955**, *27*, 1191–1194.

(2) Clearfield, A.; Stynes, J. A. The Preparation of Crystalline Zirconium Phosphate and Some Observations on Its Ion Exchange Behaviour. *Journal of Inorganic and Nuclear Chemistry* **1964**, *26*, 117–129.

(3) Yu, C.; Shao, Z.; Hou, H. A Functionalized Metal–Organic Framework Decorated with O - Groups Showing Excellent Performance for Lead(II) Removal from Aqueous Solution. *Chemical Science* **2017**, *8*, 7611–7619.

(4) Ricco, R.; Konstas, K.; J. Styles, M.; J. Richardson, J.; Babarao, R.; Suzuki, K.; Scopece, P.; Falcaro, P. Lead(Ii) Uptake by Aluminium Based Magnetic Framework Composites (MFCs) in Water. *Journal of Materials Chemistry A* **2015**, *3*, 19822–19831.

(5) Hasankola, Z. S.; Rahimi, R.; Safarifard, V. Rapid and Efficient Ultrasonic-Assisted Removal of Lead(II) in Water Using Two Copper- and Zinc-Based Metal-Organic Frameworks. *Inorganic Chemistry Communications* **2019**, *107*, 107474.

(6) Pham, H. M. N.; Phan, A. V. N.; Phan, A. N. T.; Nguyen, V. P.; Nguyen, K. M. V.; Nguyen, H. N.; Nguyen, T. M.; Nguyen, M. V. Engineering of Efficient Functionalization in a Zirconium-Hydroxyl-Based Metal–Organic Framework for an Ultra-High Adsorption of Pb2+ Ions from an Aqueous Medium: An Elucidated Uptake Mechanism. *Materials Advances* **2024**, *5*, 5118–5133.

(7) Zhu, L.; Ouyang, Q.; Liu, D.; Zhu, J.; Zhang, Y.; Zhang, J.; Shao, K.; Liu, C. Selective Capture of Pb(II) from Aqueous Solution by Mercapto Functionalized Zirconium-Based Metal Organic Frameworks. *Journal of Environmental Management* **2025**, *392*, 126642.

(8) Tang, J.; Chen, Y.; Zhao, M.; Wang, S.; Zhang, L. Phenylthiosemicarbazide-Functionalized UiO-66-NH2 as Highly Efficient Adsorbent for the Selective Removal of Lead from Aqueous Solutions. *Journal of Hazardous Materials* **2021**, *413*, 125278.

(9) Ghanbari, J.; Mobinikhaledi, A. Synthesis and Characterization of a Novel N-rich Porous Organic Polymer and Its Application as an Efficient Porous Adsorbent for the Removal of Pb(II) and Cd(II) Ions from Aqueous Solutions. *Environmental Science and Pollution Research* **2023**, *30*, 68919–68933.

(10) Melhi, S.; Alosaimi, E. H.; El-Gammal, B.; Alshahrani, W. A.; El-Aryan, Y. F.; Al-Shamiri, H. A.; Elhouichet, H. Novel Porous Organic Polymer for High-Performance Pb(II) Adsorption from Water: Synthesis, Characterization, Kinetic, and Isotherm Studies. *Crystals* **2023**, *13*, 956.

(11) Li, M.; Chen, L.; Du, J.; Gong, C.; Li, T.; Wang, J.; Li, F.; She, Y.; Jia, J. Thiol–Ene Click Reaction Modified Triazinyl-Based Covalent Organic Framework for Pb(II) Ion Effective Removal. *ACS Applied Materials & Interfaces* **2024**, *16*, 8688–8696.

(12) Zhong, J.; Cao, Y.; Zhu, J.; Wang, Y.; Yu, B.; Li, J.; Huang, J. Facile Construction of Phenolic Hydroxyl Anchored Covalent Organic Frameworks/Chitosan Composite Aerogels for Efficient Adsorption of Pb (II) from Water. *Separation and Purification Technology* **2025**, *354*, 129087.

(13) Sun, H.; Chen, X.; Wu, Z.; Ma, W.; Li, S.; Li, C.; Wu, D.; Fu, K.; Ding, K. Ortho-N-S/N-O Dual-Site Synergistic COF for Efficient Selective Removal and Highly Sensitive Detection of Hydrated Lead Ions. *Chemical Engineering Journal* **2025**, *521*, 167182.

(14) Schertenleib, T.; Schmuckler, D.; Chen, Y.; Jin, G. B.; Queen, W. L.; Billinge, S. J. L. Testing Protocols for Obtaining Reliable Pair Distribution Functions from Laboratory X-Ray Sources Using PDFgetX3. *Chemistry–Methods* **2025**, *n/a*, 2500001.
